# Active constituents of *Zanthoxylum nitidium* from Yunnan Province against leukaemia cells in vitro

**DOI:** 10.1186/s13065-021-00771-0

**Published:** 2021-07-23

**Authors:** Ying Deng, Tongtong Ding, Lulu Deng, Xiaojiang Hao, Shuzhen Mu

**Affiliations:** 1 State Key Laboratory of Functions and Applications of Medicinal Plants, Guizhou Medical University, Guiyang, 550014 China; 2 College of Pharmacy, Guizhou University, Guiyang, 550025 China; 3 Key Laboratory of Chemistry for Natural Products of Guizhou Province and Chinese Academy of Sciences, Guiyang, 550014 China

**Keywords:** *Zanthoxylum nitidium*, Alkaloids, Benzophenanthridine, Leukaemia, HEL cell, Cell cycle, Cell apoptosis

## Abstract

*Zanthoxylum nitidium* (Roxb.) DC (Rutaceae) is well known for inhibiting the proliferation of human gastric, liver, kidney and lung cancer cells, though research on its potential use in treating leukaemia is relatively rare. Twenty-six compounds were isolated from the chloroform and petroleum ether extracts of the roots and leaves of *Z. nitidium* (*Zanthoxylum nitidium*). They were ( +)-9′-*O*-transferuloyl-5, 5′-dimethoxylaricriresinol (1), 8-(3′-oxobut-1′-en-1′-yl)-5, 7-dimethoxy-coumarin (2), 5, 7, 8-trimethoxy-coumarin (3), 5-(3′, 3′-dimethyl-2′-butenyloxy)-7, 8-dimethoxy-coumarin (4), 2-(5-methoxy-2-methyl-1*H*-indol-3-yl) methyl acetate (5), 2′-(5, 6-dihydrochleletrythrine-6-yl) ethyl acetate (6), 6-acetonyldi-hydrochelerythrine (7), 6*β*-hydroxymethyldihydronitidine (8), bocconoline (9), zanthoxyline (10), *O*-methylzanthoxyline (11), rhoifoline B (12), *N*-nornitidine (13), nitidine (14), chelerythrine (15), 4-hydroxyl-7,8-dimethoxy-furoquinoline (16), dictamnine (17), *γ*-fagarine (18), skimmianine (19), robustine (20), R-( +)-platydesmine (21), 4-methoxyl-1-methyl-2-quinoline (22), 4-methoxy-2-quinolone (23), liriodenine (24), aurantiamide acetate (25), 10*-O*-demethyl-12-*O*-methylarnottianamide (26). Four among them, compounds **4 – 6** and **16**, were first confirmed in this study by UV, IR, 1D, 2D NMR and HR-ESI–MS spectra. Compounds **1 – 2** and **11** were isolated from *Z. nitidium* for the first time. Of the assayed compounds, **1**, **2**, **9**, **10**, **14**, **15** and **24,** exhibited good inhibitory activities in the leukaemia cell line HEL, whereas compound **14** (IC_50_: 3.59 *µ*M) and compound **24** (IC_50_: 15.95 *µ*M) exhibited potent inhibitory activities. So, to further investigate the possible mechanisms, cell cycle and apoptosis assays were performed, which indicated that compound **14** causes obvious S-phase arrest in HEL cells and induced apoptosis, whereas compound **24** only induced apoptosis. The present results suggested both compounds **14** and **24** are promising potential anti-leukaemia drug candidates.

**Supplementary Information:**

The online version contains supplementary material available at 10.1186/s13065-021-00771-0.

## Introduction

Leukaemia is closely related to the haematopoietic system, which includes the bone marrow [1], and malignant tumours of the haematopoietic system pose a serious threat to human health and life. Although early high-dose combination chemotherapies can achieve complete remission in many patients, the 5-year survival rate of these patients is still unsatisfactory [2], and the discovery of new anti-leukaemia drugs is very important.

Identifying candidate drug molecules in natural products is an important approach for discovering innovative drugs. *Zanthoxylum nitidium* (Roxb.) DC, locally called “liangmianzhen”, belongs to the family Rutaceae [3]. The palnt is distributed in Guangdong, Fujian, Yunnan, and Taiwan provinces of China. The chemical components of *Z. nitidium* are diverse and complex, including alkaloids, flavonoids, lignans and coumarins. Research on active substances has mainly focused on alkaloids, especially benzophenanthridine, furanquinoline, quinolones, amides, and aporphine, and a much smaller number of non-alkaloids have also been reported [4]. To data, previous studies on the biological activity of *Z. nitidium* have examined inhibition of the proliferation of human gastric, liver, kidney, lung and nasopharyngeal carcinoma cells [5]. In contrast, the anti-leukaemia properties of this plant are comparatively unknown. High expression of Fli-1 gene plays an important regulatory role in the process of vascular endothelial cell generation and tumour cell proliferation, as well as in promoting tumorigenesis and development [6, 7]. As the Fli-1 gene is a new target for drug screening, we sought to investigate the involvement of inhibitory effects on Fli-1 against leukaemia by active compounds of *Z. nitidium*.

In our previous work, ethanol extracts of *Z. nitidium* exhibited significant inhibitory effects on the proliferation of HEL cells (The human erythroleukemia lines), which highly express Fli-1, with no significant toxicity in vitro. To find a lead compound with a good effect on the Fli-1 gene, 26 compounds were isolated, purified and identified from the roots and leaves of *Z. nitidium* from Yunnan province, and their antitumour activities against HEL cells were evaluated. The chemical structures of compounds **4**, **5**, **6** and **16** were first characterized through spectroscopic analyses based on UV (Ultraviolet and visible spectrum), IR (Infrared spectroscopy), 1D and 2D NMR, and HR-ESI–MS spectra. Moreover, the antitumor activities of the 26 compounds in HEL cells were first evaluated, and the possible mechanism of two active compounds was investigated.

## Materials and methods

### Chemical reagents

INOVA-600 MHz superconducting nuclear magnetic resonance spectrometer (American Varian, TMS internal standard); HPMS5973 mass spectrometer (HP, USA); ZF-2 type three-purpose UV instrument (Shanghai Anting Electronic Instrument Factory); silica gel G (Qingdao Ocean Chemical Plant Branch) and reversed-phase silica gel C-18 (Rp-18, 40–63 m) (Amersham Biosciences, Sweden) for column chromatography; silica gel plates GF254 (Qingdao Puke Separation Material Co., Ltd.) for thin-layer chromatography; Sephadex LH-20 (Amersham Biosciences, Sweden); deuterated reagents for NMR spectroscopy (Wuhan Spectrum Company of Chinese Academy of Sciences); 5% (φ) concentrated sulfuric acid ethanol solution, an 8% (ω) phosphomolybdic acid ethanol solution, and a modified caesium iodide potassium test solution for staining TLC plates; 3111 CO_2_ incubator (Thermo Fisher Scientific Co., Ltd.); X-15R centrifuge (Backman, USA); Synergy2 multi-function microplate detector (Gene Branch Chengdu Branch); TS100 Nikon binocular inverted microscope (Shanghai Shisen Vision Technology Co., Ltd.); BD AccuriTM C6 flow cytometer (BD Biosciences); 96-well culture plates (Nisi Biotechnology Co., Ltd.); and 6-well culture plates (Nisi Biotechnology Co., Ltd.).

### Biological reagents

Human leukaemia cell line HEL (ATCC); adriamycin (Solarbio, D8740); Dulbecco's modified Eagle medium (DMEM, Gibco, C11995500CP); foetal bovine serum (FBS, Bio IND, 04–002-1A); antibiotic–antimycotic (Life Technologies, 15,240–112); bovine serum albumin (Life Technologies, 15,561,012); Cell Titer Glo (CTG, PROMEGA, G7572); flow cytometer (ACEN, NovoCyte); microplate reader (BioTek, EPOCH); annexin V and propidium iodide (PI, DOJINDO, AD10).

### Plant material

The roots and leaves of *Zanthoxylum nitidum* (Roxb.) DC. were collected in Mengla County, Xishuangbanna, Yunnan Province. The plant material was identified as *Zanthoxylum nitidium* (Roxb.) DC. by Dr. Chunfang Xiao, Xishuangbanna Tropical Botanical Garden, Chinese Academy of Sciences. The voucher plant specimen (20140408) is now in the State Key Laboratory of Functions and Applications of Medicinal Plants, Guizhou Medical University.

### Extraction and isolation

Air-dried roots and leaves of *Z. nitidum* (20.0 kg) were extracted by refluxing in 95% EtOH (100 L) three times (4, 3, and 2 h). After filtration, the combined EtOH extracts were concentrated to remove the alcohol, and the residue was resuspended in an appropriate volume of water. The mixture was extracted three times with equal volumes of petroleum ether and chloroform to afford 180.0 g of petroleum ether extract and 190.2 g of chloroform extract. The chloroform extract (190.2 g) was separated on a silica gel (50–74 μm) column eluted with a gradient of chloroform-MeOH (volume ratio: 100⁚ 1 to 0⁚ 100) to obtain 15 fractions (Fr.1 ~ Fr.15). The Fr.2 fraction was recrystallized from the chloroform-MeOH solvent to afford compound **10** (1.3 g); Fr.4 was recrystallized to afford compound **24** (360 mg). Each fraction was repeatedly subjected to normal-phase silica gel column chromatography, reversed-phase silica gel column chromatography and Sephadex LH-20 column chromatography (alternating the use of MeOH and chloroform-MeOH as the eluents) to afford compounds **1** (15 mg), **2** (49 mg), **3** (20 mg), **4** (90 mg), **5** (19 mg), **6** (5 mg), **7** (50 mg), **8** (11 mg), **9** (29 mg), **11** (22 mg), **12** (30 mg), **13** (6 mg), **14** (58 mg), **15** (7 mg), **16** (30 mg), **20** (14 mg), **21** (5 mg), **23** (22 mg), **25** (8 mg), and **26** (20 mg). The petroleum ether extract (180.0 g) was separated on a silica gel (50–74 μm) column eluted with a gradient of petroleum ether-ethyl acetate (volume ratio: 100: 1 to 0: 100) to afford 8 fractions. The same purification method was used to obtain compounds **17** (30 mg), **18** (460 mg), **19** (60 mg), and **22** (31 mg).

#### 5-(3′,3'-dimethyl-2'-butenyloxy)-6,8-dimethoxy-coumarin (4)

Yellow solid. UV (CH_3_OH) *λ* max: 206, 263 and 323 nm. ^1^H and ^13^C NMR (Table 1). HR-ESI–MS: *m/z* 313.1585 [M + Na]^+^ (calculated for C_16_H_18_O_5_).

**Table 1:** ^1^H (600 MHz) and ^13^C (151 MHz) NMR data for compound **4** in CDCl_3_

Position	*δ*_H,_ m(*J* in Hz)	*δ*_C_	HMBC
2		160.9	
3	6.16, d, (7.1)	111.0	C-8a, C-2
4	7.96, d, (7.1)	138.8	C-5a, C-2, C-5
5		128.8	
6	6.33, d (1.5)	91.3	C-8a, C-5, C-8, C-7
7		156.6	
8		152.3	
8a		103.9	
5a		149.0	
1′	4.54, dd (7.5, 1.5)	70.0	C-2′, C-5, C-3′
2′	5.57, d (1.5)	120.2	C-4′, C-5′
3′		139.0	
4′	1.68, s	18.0	C-5′, C-2′, C-3′
5′	1.73, s	25.8	C-4′, C-2′, C-3′
7-OCH_3_	3.94, s	56.4	C-7
8-OCH_3_	3.90, s	56.4	C-8

#### 2-(5-methoxy-2-methyl-1H-indol-3-yl) methyl acetate (5)

Tawny oil. UV (CH_3_OH) *λ* max: 218 and 279 nm. ^1^H and ^13^C NMR (Table 2). HR-ESI–MS: *m/z* 234.1124 [M + H]^+^ (calculated for C_13_H_15_O_3_N).

**Table 2:** ^1^H (600 MHz) and ^13^C (151 MHz) NMR data for compound **5** in CDCl_3_

Position	*δ*_H,_ m(*J* in Hz)	*δ*_C_	HMBC
2		132.8	
3		128.9	
4	7.04, m	111.1	C-5, C-3, C-7
5		154.1	
6	6.75, dd (8.7, 2.4)	110.8	C-7, C-5, C-7a
7	6.98, d (8.7)	100.4	C-7a, C-5, C-6, C-4, C-4a
4a		104.1	
7a		130.2	
8	3.65, s	30.3	C-2, C-3, C-4a
9		172.8	
10	2.28, s	11.7	C-2
5-OCH_3_	3.84, s	56.0	C-5
9-OCH_3_	3.65, s	52.0	C-9

#### 2′-(5,6-dihydrochleletrythrine-6-yl) ethyl acetate (6)

Yellow oil. UV (CH_3_OH) *λ* max: 201, 283 and 224 nm. ^1^H and ^13^C NMR (Table 3). HR-ESI–MS: *m/z* 436.1752 [M + H]^+^ ((calculated for C_14_H_13_O_4_N).

**Table 3:** ^1^H (600 MHz) and ^13^C (151 MHz) NMR data for compound **6** in CDCl_3_

Position	*δ*_H,_ m(*J* in Hz)	*δ*_C_	HMBC
1	7.12, s	104.3	C-2, C-12a, C-12
2		148.0	
3		147.5	
4	7.57, s	101.0	C-3, C-4b
4a		131.1	
4b		139.3	
6	5.02, m	55.1	C-4b, C-10a
6a		128.0	
7		145.5	
8		152.1	
9	6.99, d (*J* = 8.5 Hz)	111.6	C-7, C-10a
10	7.58, d (*J* = 8.5 Hz)	118.8	C-8, C-10b, C-6a
10a		124.9	
10b		123.8	
11	7.73, d (*J* = 8.7 Hz)	119.8	C-4b, C-4a, C-10a
12	7.50, d (*J* = 8.7 Hz)	124.0	C-1, C-10b, C-12a
12a		127.5	
N-CH_3_	2.68, s	42.9	C-6
7-OCH_3_	3.99, s	61.0	C-7
8-OCH_3_	3.95, s	55.8	C-8
-O-CH_2_-O-	6.06, s	101.0	
1′		171.7	
2′	2.38, s	39.2	C-1′, C-6
3′	4.17, d (*J* = 7.1 Hz)	60.3	
4′	1.21, d (*J* = 7.1 Hz)	14.2	C-3′

#### 4-hydroxyl-7, 8-dimethoxy-furoquinoline (16)

Tawny solid. UV (CH_3_OH) *λ* max: 249, 201 and 316 nm. ^1^H and ^13^C NMR (Table 4). HR-ESI–MS: *m/z* 246.0760 [M + H]^+^ (calculated for C_13_H_12_O_4_N).

**Table 4:** ^1^H (600 MHz) and ^13^C (151 MHz) NMR data for compound **16** in Pyridine-*d*_5_

Position	*δ*_H,_ m(*J* in Hz)	*δ*_C_	HMBC
2		164.5	
3		101.6	
4		142.3	
4a		114.1	
5	8.13, d (9.1)	118.8	C-4, C-8, C-8a
6	7.54, d (9.1)	117.3	C-7, C-8, C-4a
7		140.2	
8		151.6	
8a		157.4	
3b	7.15, d (2.7)	105.3	C-2, C-3, C-4
2a	7.80, d (2.7)	142.9	C-2, C-3, C-3b
7-OCH_3_	4.23, s	61.1	C-7
8-OCH_3_	4.27, s	58.9	C-8
-OH	12.03, s		

### CAS numbers

5, 7, 8-trimethoxy-coumarin (**3,** 60796–65-8), 2-(5-methoxy-2-methyl-1*H*-indol-3-yl) methyl acetate (**5,** 7588–36-5), 6-acetonyldi-hydrochelerythrine (**7**, 15575–49-2), bocconoline (**9**, 112025–60-2), zanthoxyline (**10**, 54354–62-0), *O*-methylzanthoxyline (**11**, 6900–99-8), nitidine (**14**, 13063–04-2), chelerythrine (**15**, 34316–15-9), dictamnine (**17**, 484–29-7), *γ*-fagarine (**18**, 524–15-2), skimmianine (**19**, 83–98-4), robustine (**20**, 2255–50-7), R-( +)-platydesmine (**21**, 7764–73-0), 4-methoxy-2-quinolone (**23**, 27667–34-1), liriodenine (**24**, 475–75-2), aurantiamide acetate (**25**, 56121–42-7), and 10*-O*-demethyl-12-*O*-methylarnottianamide (**26**, 1234313–87-1).

### CTG assay for antitumour activity

The human leukaemia cell line HEL was purchased from American Type Culture Collection, and the cells were cultured in DMEM. All media were supplemented with 10% foetal bovine serum (FBS), 100 units/mL penicillin, and 100 units/mL streptomycin (Invitrogen). The cells were cultured at 37 °C in a humidified environment with 5% CO_2_ and passaged once every 2 days for three generations. The cells were incubated in fresh cell culture medium and washed carefully to avoid false-positive results. Briefly, HEL cells (8 × 10^3^ cells per well) were seeded into 96-well plates, and the plates were incubated for 24 h. Then, 10 *μ*L of adriamycin were added as a positive control, and 10 *μ*L of various concentrations (40, 20, 10, 5, 2.5, 1.25 *μ*M) of compounds (5 × 10^–6^ mol/L) were added as the test group, with 5 wells per group. After incubation for 72 h, 20 *μ*L of CTG reagent were added, and the cells were incubated for 10 min. After centrifugation (1500 rpm, 15 min) the supernatant was poured off, 160 *μ*L of DMSO were added to each well, and the plate was heated and shaken for 10 min. Finally, the chemiluminescence of each well were determined using a microplate reader. After the experiment were repeated three times, the IC_50_ value was calculated from the curves generated by plotting the percentage of viable cells versus the tested concentration on a logarithmic scale using Sigma Plot 10.0 software.

### Cell apoptosis analysis

Apoptosis was detected by flow cytometry using Annexin V-FITC according to the manufacturer's protocol (BD Biosciences). HEL cells were treated with compounds **14** and **24** for 36 h before Annexin V and propidium iodide staining. The cells were kept under dark conditions at room temperature for 15 min before being subjected to flow cytometry analysis.

### Cell cycle analysis

Cell cycle analysis was conducted by propidium iodide (PI) staining after treatment with compounds **14** and **24** for 36 h. Briefly, cells were plated in culture dishes and cultured with prepared DMEM medium for 12 h, after which the cells were treated with compounds **14** and **24** for 36 h, and the supernatant was removed. The treated cells were fixed with 70% ethanol overnight before staining with PI mixed with RNase. The cells were kept under dark conditions at room temperature for 30 min before being subjected to flow cytometry analysis.

### Statistical analysis

All measurements were made in triplicate, and all data are expressed as the means ± SEM of three independent experiments. Significant differences from the respective control for each experimental group were examined by one-way analysis of variance (ANOVA) using GraphPad Prism 5 software. P < 0.05 was considered statistically significant.

## Results and discussion

### Isolation and structural elucidation

**Fig. 1:**
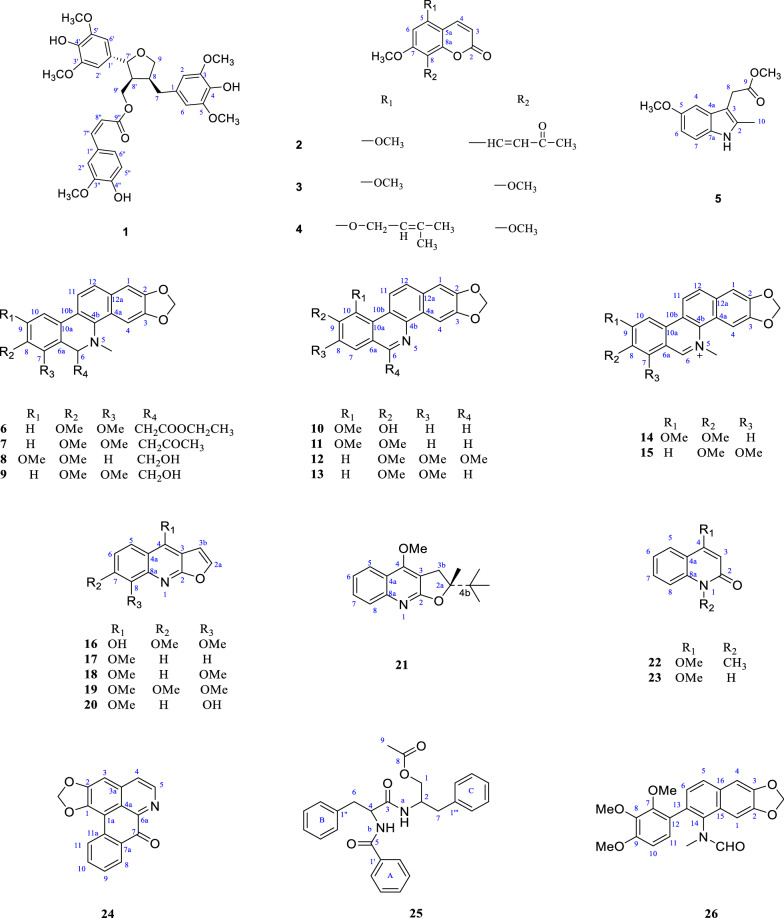
Compounds **1 – 26** isolated from the roots and leaves *Zanthoxylum nitidium*

Dried roots and leaves (20 kg) of *Z. nitidium* were heated and refluxed in 95% EtOH. The resulting extract was concentrated and then partitioned between petroleum ether and chloroform. The extracts were further separated by recrystallization and various forms of column chromatography (CC) to afford compounds **1 – 26** (Fig. 1).

### Chemical structure of compound 4

Compound **4** was obtained as yellow solid with a molecular formula of C_16_H_18_O_5_ deduced from its HR-ESI–MS spectrum (*m/z* 291.1585 [M + H]^+^). The UV profile of **4** displayed the λ max values of 206, 263 and 323 nm, and its IR spectrum showed absorptions representing a lactone ring (1726 cm^−1^) and an aromatic ring (1502 and 1432 cm^−1^). The above data indicated that compound **4** contains a lactone ring. The ^1^H-NMR data (Table 1) showed the following: three aromatic proton signals [*δ*_H_ 7.96 (d, *J* = 7.1 Hz, 1H), 6.16 (d, *J* = 7.1 Hz, 1H), and 6.33 (d, *J* = 1.5 Hz, 1H)]; two methoxyl moieties [*δ*_H_ 3.94 (s, 3H) and 3.90 (s, 3H)]; two methyl [*δ*_H_ 1.68 (s, 3H) and 1.73 (s, 3H)]; and one methylene [*δ*_H_ 4.54 (dd, *J* = 7.5, 1.5 Hz, 2H)]. The above nuclear magnetic resonance data are similar to those reported for compound **4′** in the literature [8, 9].

**Fig. 2:**
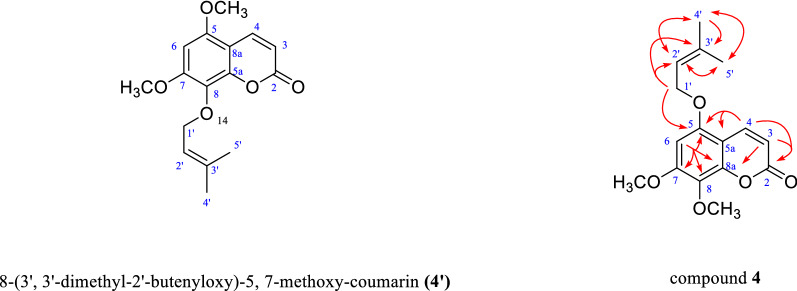
The structure of compound **4ʹ** and HMBC correlations of compound **4**

A previous report [8] suggested the carbon signals of the C-8 and C-5 of compound **4'** were slightly distinct with compound **4**. Therefore, we speculate that the different carbon chemical shift at C-8 and C-5 may be caused by 3', 3'-dimethyl-2'-butenyloxy group positions. As illustrated in Fig. 2, HMBC correlations of the protons H-1' (*δ*_H_ 4.54) with C-2′ (*δ*_C_ 120.2), C-3′ (*δ*_C_ 139.0), and C-5 (*δ*_C_ 128.8) indicated that the 3', 3'-dimethyl-2'-butenyloxy group of compound **4** is attached at the C-5 position. HMBC correlations of H-4 (*δ*_H_ 7.96) to C-5a (*δ*_C_ 149.0), C-2 (*δ*_C_ 160.9) and C-5 (*δ*_C_ 128.8); H-3 (*δ*_H_ 6.16) to C-8a (*δ*_C_ 103.9) and C-2 (*δ*_C_ 160.9) indicated that the lactone ring is close to C-8. Finally, the proton chemical shift for 7-OCH_3_ (*δ*_H_ 3.94, s), as based on HMBC data, correlates with the C-7 (*δ*_C_ 156.6), and the signal for 8-OCH_3_ (*δ*_H_ 3.90, s) correlates with the C-8 (*δ*_C_ 152.3). The two -OCH_3_ groups are at C-7 and C-8. The above nuclear magnetic resonance data indicated that compound **4** is consistent with 5-(3', 3'-dimethyl-2'-butenyloxy)*-*7, 8-methoxy-coumarin, which has been previously reported in the literature [10]. As the ^13^C-NMR data of compound **4** were not assigned in the literature, its 1D and 2D NMR data were analyzed in this study.

### Chemical structure of compound 5

Compound **5** was isolated as a tawny oil. Its molecular formula was determined to be C_13_H_15_O_3_N based on its positive HR-ESI–MS data (*m/z* 234.1124 [M + H]^+^). The UV profile of **5** displayed the λ max values at 218 and 279 nm, and the IR spectrum showed absorptions for an *α*, *β*-unsaturated ester carbonyl (1731 cm^−1^) and an aromatic ring (1593 and 1430 cm^−1^). According to the ^1^H-NMR data in Table 2, there are three aromatic protons chemical shift [*δ*_H_ 7.04 (m, 1H), 6.75 (dd, *J* = 8.7, 2.4 Hz, 1H), and 6.98 (d, *J* = 8.7 Hz, 1H)], a methylene moiety [*δ*_H_ 3.65 (s, 2H)], and two methoxy [*δ*_H_ 3.84 (s, 3H) and 3.65 (s, 3H)]. The above nuclear magnetic resonance data indicated that compound **5** is consistent with 2-(5-methoxy-2-methyl-1*H*-indol-3-yl) methyl acetate, which has been previously reported in the literature [11].

**Fig. 3:**
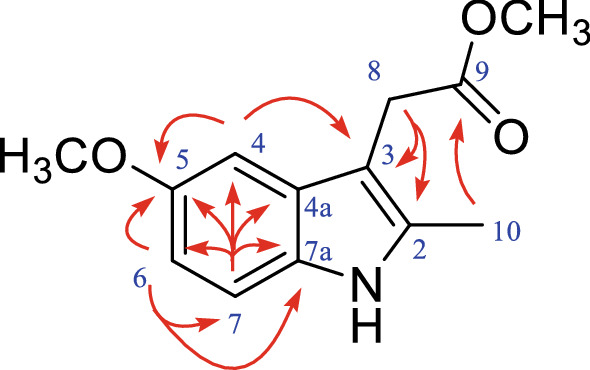
HMBC correlations of compound **5**

Similar to compound **4**, the ^13^C-NMR data for compound **5** was not reported in the previous literature, and the 1D and 2D NMR data were thus analysed. As depicted in Table 2, the coupling constant of the proton chemical shift at H-6 (*δ*_H_ 6.75) and H-7 (*δ*_H_ 6.98) is *J* = 8.7 Hz, suggesting that the two proton signals are ortho-coupled to the benzene ring. The three protons at *δ*_H_ 7.04 (m, 1H), *δ*_H_ 6.75 (dd, *J* = 8.7, 2.4 Hz, 1H), *δ*_H_ 6.98 (d, *J* = 8.7 Hz, 1H) correlated with carbons at *δ*_C_ 111.1, 110.8 and 100.4 in HSQC spetrum, respectively, indicated an aromatic ring. At the same time, the HMBC data (Fig. 3) showed correlations of H-8 (*δ*_H_ 3.65) with C-2 (*δ*_C_ 132.8), C-3 (*δ*_C_ 128.9), and C-4a (*δ*_C_ 104.1), suggesting that the compound contains an indole moiety; and of H-10 (*δ*_H_ 2.28) with C-2 (*δ*_C_ 132.8), suggesting the presencen of a methyl acetate. Finally, the HMBC data revealed a correlation of 5-OCH_3_ (*δ*_H_ 3.84, s) with C-5 (*δ*_C_ 154.1) and of 9-OCH_3_ (*δ*_H_ 3.65, s) with C-9 (*δ*_C_ 172.8). These results indicated that the two -OCH_3_ groups are at C-5 and C-9. Compound **5** was thus named 2-(5-methoxy-2-methyl-1*H*-indol-3-yl) methyl acetate.

### Chemical structure of compound 6

Compound **6** was isolated as a yellow oil. Its molecular formula was determined to be C_25_H_25_O_6_N based on its positive HR-ESI–MS data (*m/z* 436.1752 [M + H]^+^). The UV profile of **6** revealed λ max values of 201, 283 and 224 nm and its IR spectrum showed absorption bands for an *α*, *β*-unsaturated ester carbonyl (1736 cm^−1^) and an aromatic ring (1492 and 1463 cm^−1^). The ^1^H-NMR (Table 3) spectrum of compound **6** showed signals characteristic for two pairs of aromatic protons chemical shift [*δ*_H_ 7.73 (d, *J* = 8.7 Hz, 1H) and 7.50 (d, *J* = 8.7 Hz, 1H), 6.99 (d, *J* = 8.5 Hz, 1H) and 7.58 (d, *J* = 8.5 Hz, 1H)], two aromatic proton signals [*δ*_H_ 7.57 (s, 1H) and 7.12 (s, 1H)], two groups of methyl [*δ*_H_ 2.68 (s, 3H) and 1.21 (dd, *J* = 7.1 Hz, 3H)], three methylene moieties [*δ*_H_ 6.06 (s, 2H), 2.38 (s, 2H) and 4.17 (d, *J* = 7.1 Hz, 2H)], and two methoxy [*δ*_H_ 3.99 (s, 3H) and 3.95 (s, 3H)]. Compound **6** is a benzophenanthridine alkaloids based on the above nuclear magnetic resonance data. We found compound **6** to be consistent with 2'-(5, 6-dihydrochleletrythrine-6-yl) ethyl acetate, which has been previously reported in the literature [12].

**Fig. 4:**
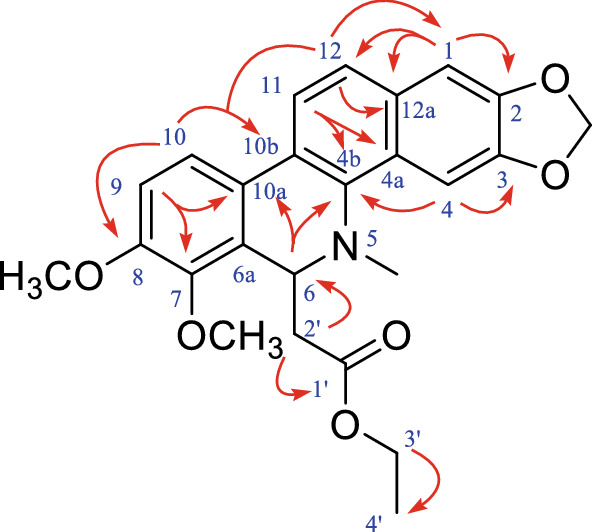
HMBC correlations of compound **6**

The NMR data for compound **6** were assigned for the first time according to its 2D-NMR data. From the ^1^H-NMR data in Table 3, the coupling constant between the proton signals at H-11 (*δ*_H_ 7.73) and H-12 (*δ*_H_ 7.50) is *J* = 8.7 Hz, and that between H-9 (*δ*_H_ 6.99) and H-10 (*δ*_H_ 7.58) is *J* = 8.5 Hz, indicating that the two pairs of protons chemical shift are ortho-coupled to the phenyl ring. As depicted in Fig. 4, HMBC data exhibited correlations of H-1 (*δ*_H_ 7.12) with C-2 (*δ*_C_ 148.0), C-12 (*δ*_C_ 124.0), and C-12a (*δ*_C_ 127.5) and of H-4 (*δ*_H_ 7.57) with C-3 (*δ*_C_ 147.5) and C-4b (*δ*_C_ 139.3), indicating that compound **6** is a benzophenanthrene derivative. The direct HSQC (Figure S19, Additional file 1) correlations between H-6 (δ_H_ 4.95) and C-6 (δ_C_ 55.1) also demonstrated that compound **6** is a chelerythrine. Similarly, based on the HMBC (Fig. 4), the correlations of H-2′ (*δ*_H_ 2.38) with C-2 (*δ*_C_ 148.0), C-1′ (*δ*_C_ 171.7), and C-6 (*δ*_C_ 55.1) and of H-4′ (*δ*_H_ 1.21) with C-3′ (*δ*_C_ 60.3) suggested the presence of an ethyl acetate group. Finally, the HMBC correlations of 7-OCH_3_ (*δ*_H_ 3.99) with C-7 (*δ*_C_ 145.5) and of 8-OCH_3_ (*δ*_H_ 3.95) with C-8 (*δ*_C_ 152.1) indicated that the two -OCH_3_ groups are at C-7 and C-8.

### Chemical Structure of compound 16

Compound **16** was obtained as tawny solid with a molecular formula of C_13_H_11_O_4_N deduced from its HR-ESI–MS spectrum (*m/z* 246.0760 [M + H]^+^). The UV profile of **16** revealed λ max values of 249, 201 and 316 nm, which are similar to those of quinoline [11]. The IR spectrum displayed absorption bands for an aromatic ring (1516 and 1443 cm^−1^) and an ether (1151 and 1046 cm^−1^). As indicated in Table 4, ^1^H-NMR detected two pairs of aromatic proton signals [*δ*_H_ 8.13 (d, *J* = 9.1 Hz, 1H) and 7.54 (d, *J* = 9.1 Hz, 1H), 7.15 (d, *J* = 2.7 Hz, 1H) and 7.80 (d, *J* = 2.7 Hz, 1H)], two methoxy moieties [*δ*_H_ 4.23 (s, 3H) and 4.27 (s, 3H)], and an active hydrogen chemical shift [*δ*_H_ 12.03 (s, 1H)]. Based on the above nuclear magnetic resonance data, compound **16** is consistent with 4-hydroxy-7, 8-dimethoxy-furoquinoline, which has been previously reported in the literature [14].

**Fig. 5:**
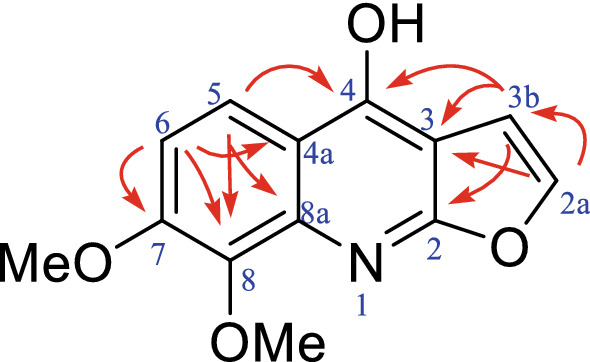
HMBC correlations of compound **16**

To clarify the structure of **16**, we for the first time assigned its NMR data. The ^1^H-NMR data (Table 4), showed a coupling constant between the chemical shift at H-5 (*δ*_H_ 8.13) and H-6 (*δ*_H_ 7.54) is *J* = 9.1 Hz; these two proton signals are ortho-coupled to the phenyl ring. The HMBC data in Fig. 5 illustrate the correlations of H-5 (*δ*_H_ 8.13) with C-4 (*δ*_C_ 142.3), C-8 (*δ*_C_ 151.6), and C-8a (*δ*_C_ 157.4) and of H-6 (*δ*_H_ 7.54) with C-6 (*δ*_C_ 117.3), C-8 (*δ*_C_ 151.6), and C-4a (*δ*_C_ 114.1), suggesting that compound **16** contains a quinoline ring. Similarly, the coupling constant between the chemical shift at H-3b (*δ*_H_ 7.15) and H-2a (*δ*_H_ 7.80) is *J* = 2.7 Hz, indicating that the protons are ortho-coupled to a furan ring. According to the HMBC data in Fig. 5, correlations of H-3b (*δ*_H_ 7.15) with C-2 (*δ*_C_ 164.5), C-3 (*δ*_C_ 101.6), and C-4 (*δ*_C_ 142.3) and of H-2a (*δ*_H_ 7.80) with C-2 (*δ*_C_ 164.5), C-3 (*δ*_C_ 101.6), and C-3b (*δ*_C_ 105.3) suggest that this compound is a furan derivative. Finally, HMBC correlations of 7-OCH_3_ (*δ*_H_ 4.23) with C-7 (*δ*_C_ 140.2) and of 8-OCH_3_ (*δ*_H_ 4.27) with C-8 (*δ*_C_ 151.6) were observed. These results indicated that the two -OCH_3_ groups are located at C-7 and C-8. The above nuclear magnetic resonance data showed that compound **16** is consistent with 4-hydroxy-7, 8-dimethoxy-furoquinoline, which has been previously reported in the literature [14], though no 1D and 2D NMR data were reported. Herein, its NMR data of compound **16** were also assigned in the present study.

Overall, twenty-two compounds (compounds **5**–**26**) were found to be alkaloids; the other four (compounds **1**–**4**) were considered to be false-positive non-alkaloids based on the modified potassium caesium iodide test, as proven based on ^1^H-NMR and ^13^C-NMR spectra. In addition, by the comparison of NMR data with those described in the literature, the 26 compounds were identified as ( +)-9′-*O*-transferuloyl-5, 5′-dimethoxylaricriresinol (**1**) [15], 8-(3′-oxobut-1′-en-1′-yl)-5, 7-dmethoxy-coumarin (**2**) [16], 5, 7, 8-trimethoxy-coumarin (**3**) [17], 5-(3′, 3′-dimethyl-2′-butenyloxy)-7, 8-dimethoxy-coumarin (**4**), 2-(5-methoxy-2-methyl-1*H*-indol-3-yl) methyl acetate (**5**), 2′-(5, 6-dihydrochleletrythrine-6-yl) ethyl acetate (**6**), 6-acetonyldi-hydrochelerythrine (**7**) [18], 6*β*-hydroxymethyldihydronitidine (**8**) [19], bocconoline (**9**) [20], zanthoxyline (**10**) [21], *O*-methylzanthoxyline (**11**) [22], rhoifoline B (**12**) [23], *N*-nornitidine (**13**) [24], nitidine (**14**) [25], chelerythrine (**15**) [26], 4-hydroxyl-7, 8-dimethoxy-furoquinoline (**16**), dictamnine (**17**) [27], *γ*-fagarine (**18**) [28], skimmianine (**19**) [13], robustine (**20**) [27], R-( +)-platydesmine (**21**) [29], 4-methoxyl-1-methyl-2-quinoline (**22**) [28], 4-methoxy-2-quinolone (**23**) [30], liriodenine (**24**) [31], aurantiamide acetate (**25**) [32], and 10*-O*-demethyl-12-*O*-methylarnottianamide (**26**) [33].

### Biological activities of the isolated compounds

To analyse the effects of the 26 compounds on leukaemia cells (HEL cell lines), their IC_50_ values against HEL cells proliferation were determined by the CTG method, using adriamycin (IC_50_: 0.021 *µ*M) as a positive control. As presented in Table 5, compound **14** (IC_50_: 3.59 *µ*M) and compound **9** (IC_50_: 7.65 *µ*M) showed the most potent inhibitory activities against HEL cells, compounds **15** (IC_50_: 15.52 *µ*M) and **24** (IC_50_: 15.95 *µ*M) exhibited moderate inhibitory activities against HEL cells. As the structures of compound **14** and compound **24** differ, different compounds of *Z. nitidium* may have inhibitory activity in HEL cells.

**Table 5:** Inhibitory activity of compounds **1**–**26** in HEL cell lines

Compounds	IC_50_ (*µ*M) ± SD	Compounds	IC_50_ (*µ*M) ± SD
1	28.84 ± 1.53	14	3.59 ± 0.82
2	22.43 ± 1.86	15	15.52 ± 0.26
3	> 30	16	> 30
4	> 30	17	> 30
5	> 30	18	> 30
6	> 30	19	> 30
7	> 30	20	> 30
8	> 30	21	> 30
9	7.65 ± 0.11	22	> 30
10	24.94 ± 1.99	23	> 30
11	> 30	24	15.95 ± 2.33
12	> 30	25	> 30
13	> 30	26	> 30
DOX	0.021 ± 1.25		

### Compounds 14 and 24 induced cell cycle arrest

**Fig. 6:**
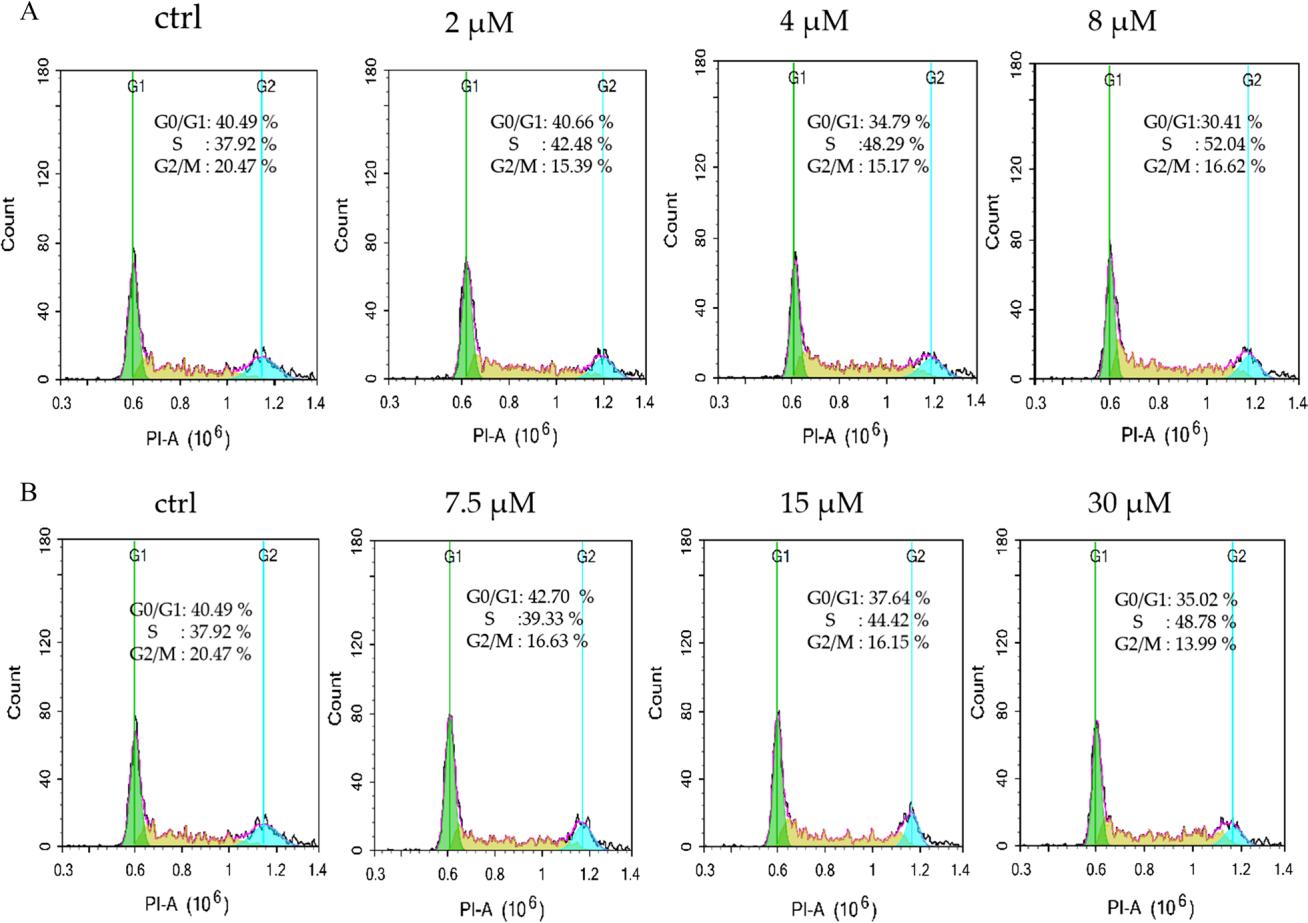
**A** Compound **14** induced cell cycle arrest at the phase. Compound **14** altered cell cycle distribution in HEL cells. Cells were exposed to DMSO or compound **14** at indicated concentrations for 36 h and then were collected for DNA content analysis by flow cytometric analysis as experiment. **B** Compound **24** induced cell cycle arrest at the phase. Compound **24** altered cell cycle distribution in HEL cells. Cells were exposed to DMSO or compound **24** at indicated concentrations for 36 h and then were collected for DNA content analysis by flow cytometric analysis as experiment

To confirm the effects of compounds **14** and **24** with different structures on the cell cycle, the cell cycle distribution of HEL cells was examined after treatment with the compounds for 36 h. As illustrated in Fig. 6, significant S-transition arrest was observed in HEL cells treated with compound **14**, which provided the most significant effect. Indeed, the fraction of cells in the S-phase was dose-dependently increased by treatment with **14**, and the population of cells in S-phase was markedly increased to 52.04% in cells treated with 8 μM compared to 37.92% in untreated cells. Conversely, compound **24**, with a different structure, had no obvious effect on the HEL cell cycle.

### Compounds 14 and 24 induced apoptosis of HEL cells

**Fig. 7:**
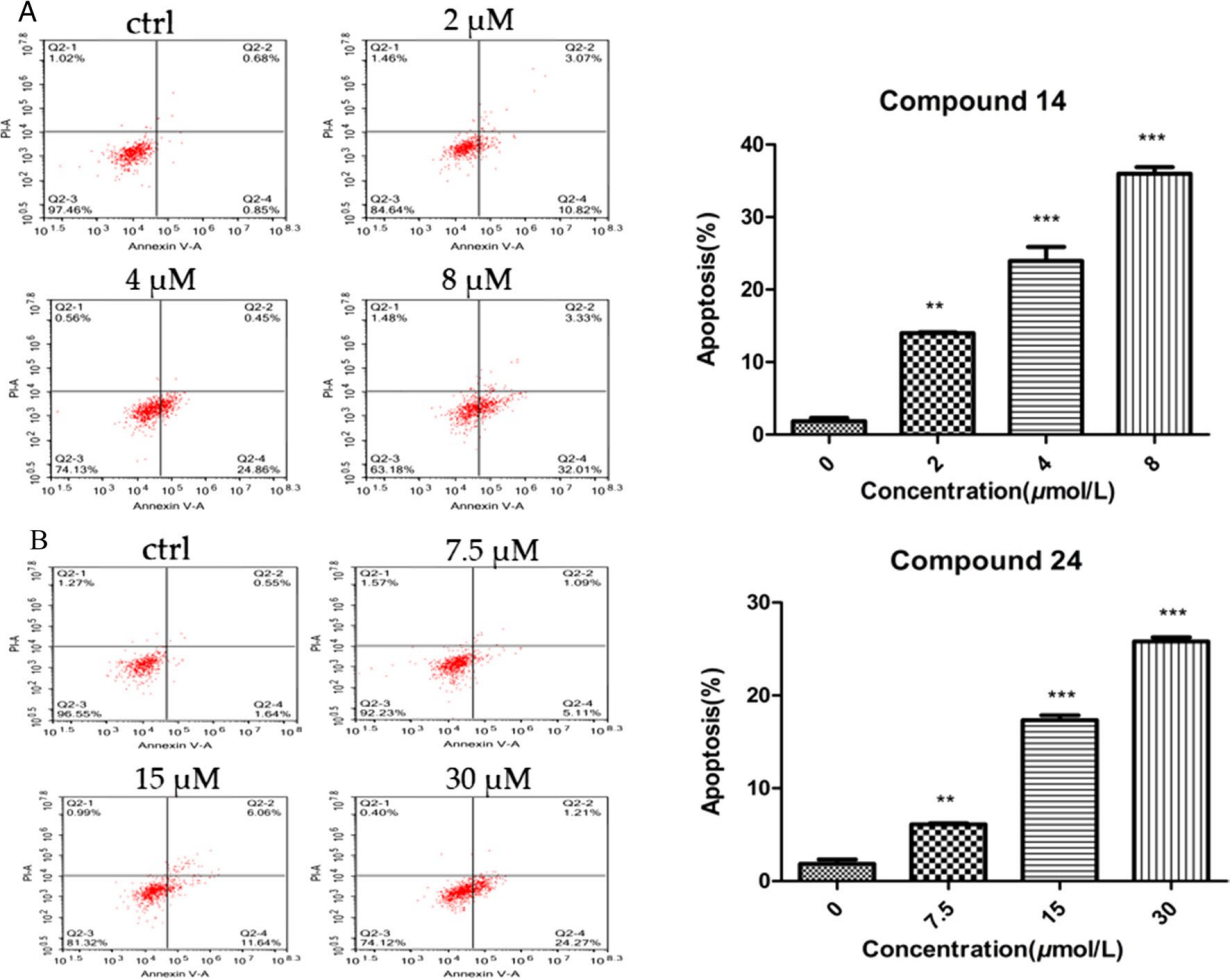
**A** Compound **14** induced apoptosis in HEL cells. Cell apoptosis was analyzed by flow cytometric analysis after Annexin V-FITC/PI staining. Cells were collected and centrifuged at 1500 rpm for 10 min after compound **14** treatment at the indicated concentrations for 36 h. **B** Compound **24** induced apoptosis in HEL cells. Cell apoptosis was analyzed by flow cytometric analysis after Annexin V-FITC/PI staining. Cells were collected and centrifuged at 1500 rpm for 10 min after compound **24** treatment at the indicated concentrations for 36 h. The changes in corresponding protein expression levels were quantified using Image J. Each bar represents the mean ± SEM (n = 3). P < 0.05, **P < 0.01 or ***P < 0.001 was considered statistically significant compared with the corresponding control values

To determine whether the antiproliferative activity of **14** and **24** is accompanied by enhanced leukaemia cell apoptosis, flow cytometry and an Annexin V-FITC apoptosis detection kit were used to detect apoptosis. Compared with untreated cells, cells treated with compounds **14** and **24** displayed significant dose-dependent increases, as shown in Fig. 7. At the same time, compound **24** at 7.5 *μ*M and 15.0 μM induced significant increases in apoptosis compared with the control group (DMSO). Compound **24** at concentrations of 7.5, 15 and 30 *μ*M promoted apoptosis from 6.11% and 17.34% to 25.81% in a dose-dependent manner. Hence, compounds **14** and **24** caused obvious apoptosis in HEL cells in a concentration-dependent manner.

## Conclusions

In summary, four compounds (**4 **– **6** and **16**) with incomplete spectra and 22 known compounds were isolated and identified from the chloroform and petroleum ether extracts of the roots and leaves of *Z. nitidium*. The chemical structures of compounds **4 **– **6** and **16** were elucidated by thorough spectroscopic analyses, and compounds **1**, **2** and **11** were isolated from *Z. nitidium* for the first time. Among the isolated compounds, **1**, **2**, **9**, **10**, **14**, **15** and **24,** which are alkaloids, exhibited good inhibitory activities in the leukaemia cell line HEL, whereas compound **14** (IC_50_: 3.59 *µ*M) and compound **24** (IC_50_: 15.95 *µ*M) exhibited potent inhibitory activities. To clarify the effect of different compound structure **14** and **24** in HEL cells, apoptosis and cell cycle assays showed that compound **14** possesses antiproliferative activity, and induces S-phase cell cycle arrest and apoptosis in HEL cells. In contrast, compound **24** only induced apoptosis in HEL cells. These results indicated that benzophenanthridine alkaloids had significant inhibition activities in leukaemia cells, providing new ideas for the structural modification and mechanism involved. It was worth mentioning that two compounds (**14** and **24**) were firstly found as the potential lead compounds with a good effect on the Fli-1 gene in leukaemia.

## Supplementary Information

**Additional file 1:** The following are available online. 1H-NMR, 13C-NMR, DEPT, HSQC, HMBC, 1H-1H-COSY, HR-ESI-MS, infrared, and ultraviolet-visible spectra of compounds 4, 5, 6 and 16.

## Data Availability

The datasets generated and/or analysed during the current study are not publicly available due [some of the datasets involved needs to be used for unpublished patents] but are available from the corresponding author on reasonable request. We have presented all our main data in the form of tables, figures and supplementary.

## Abbreviations

*Z. nitidium*: Zanthoxylum nitidium
HEL: The human erythroleukemia lines
UV: Ultraviolet and visible spectrim
IR: Infrared spectroscopy
^1^H-NMR: Proton nuclear magnetic resonance
^13^C-NMR: Carbon: nuclear magnetic resonance
HMBC: Heteronuclear multiple bond correlation
HSQC: Heteronuclear single quantum coherence
^1^H-^1^H COSY: Homonuclear chemical shift Correlation Spectroscopy
DMSO: Deuterated dimethyl sulfoxide
DMEM: Dulbecco's modified Eagle medium
FBS: Foetal bovine serum
CTG: Cell Titer Glo
PI: Annexin V and propidium iodide
