# Supplementary material for: Active constituents of Zanthoxylum nitidium from Yunnan Province against leukaemia cells in vitro
Source: BMC Chem. 2021 Jul 23;15(1):44. doi: 10.1186/s13065-021-00771-0 (PMC8305521; doi:10.1186/s13065-021-00771-0)
Supplement: Supplementary file 1 — Additional file 1: The following are available online. 1H-NMR, 13C-NMR, DEPT, HSQC, HMBC, 1H-1H-COSY, HR-ESI-MS, infrared, and ultraviolet-visible spectra of compounds 4, 5, 6 and 16. [file 13065_2021_771_MOESM1_ESM.docx]

Supplementary file,

Active constituents of *Zanthoxylum nitidum* from Yunnan Province against leukaemia cells in vitro

**Ying Deng^1, 2, 3^, Tongtong Ding^1^, Lulu Deng^1, 3^, Xiaojiang Hao^1, 3^, Shuzhen Mu^1, 3*^**

^1^ State Key Laboratory of Functions and Applications of Medicinal Plants, Guizhou Medical University, Guiyang 550014, China.

^2^ College of Pharmacy, Guizhou University, Guiyang 550025, China.

^3^ Key Laboratory of Chemistry for Natural Products of Guizhou Province and Chinese Academy of Sciences, Guiyang 550002, China.

^*^ Correspondence: e-mail: muzi0558@126.com; Tel.: 13765801160

**Supporting Information List of Contents 1**

| **No.** | **Content** | **Page** |
| --- | --- | --- |
| 1 | **Fig S1.** 1H-NMR spectrum of compound **4** in CDCl3. | 3 |
| 2 | **FigS2.** 13C-NMR spectrum and DEPT of compound 4 in CDCl3. | 3 |
| 3 | **Fig S3.** HSQC spectrum of compound 4 in CDCl3. | 4 |
| 4 | **Fig S4.** HMBC spectrum of compound 4 in CDCl3. | 4 |
| 5 | **Fig S5.** 1H- 1H COSY spectrum of compound 4 in in CDCl3. | 5 |
| 6 | **Fig S6.** IR spectrum of compound 4. | 5 |
| 7 | **Fig S7.** HR-ESI-MS spectrum of compound 4. | 6 |
| 8 | **Fig S8.** UV-Vis spectrum of compound 4 in CH3OH. | 6 |
| 9 | **Fig S9.** 1H-NMR spectrum of compound 5 in CDCl3. | 7 |
| 10 | **Fig S10.** 13C-NMR spectrum and DEPT of compound 5 in CDCl3. | 7 |
| 11 | **Fig S11.** HSQC spectrum of compound 5 in CDCl3. | 8 |
| 12 | **Fig S12.** HMBC spectrum of compound 5 in CDCl3. | 8 |
| 13 | **Fig S13.** 1H- 1H COSY spectrum of compound 5 in CDCl3. | 9 |
| 14 | **Fig S14.** IR spectrum of compound 5. | 9 |
| 15 | **Fig S15.** HR-ESI-MS spectrum of compound 5. | 10 |
| 16 | **Fig S16.** UV-Vis spectrum of compound 5 in CH3OH. | 10 |
| 17 | **Fig S17.** 1H-NMR spectrum of compound 6 in CDCl3. | 11 |
| 18 | **Fig S18.** 13C-NMR spectrum and DEPT of compound 6 in CDCl3. | 11 |
| 19 | **Fig S19.** HSQC spectrum of compound 6 in CDCl3. | 12 |
| 20 | **Fig S20.** HMBC spectrum of compound 6 in CDCl3. | 12 |
| 21 | **Fig S21.** 1H- 1H COSY spectrum of compound 6 in CDCl3. | 13 |
| 22 | **Fig S22.** IR spectrum of compound 6. | 13 |
| 23 | **Fig S23.** HR-ESI-MS spectrum of compound 6. | 14 |
| 24 | **Fig S24.** UV-Vis spectrum of compound 6 in CH3OH. | 14 |
| 25 | **Fig S25.** 1H-NMR spectrum of compound 16 in Pyridine-d5. | 15 |
| 26 | **Fig S26.** 13C-NMR spectrum and DEPT of compound 16 in Pyridine-d5. | 15 |
| 27 | **Fig S27.** HSQC spectrum of compound 16 in Pyridine-d5. | 16 |
| 28 | **Fig S28.** HMBC spectrum of compound 16 in Pyridine-d5. | 16 |
| 29 | **Fig S29.** 1H- 1H COSY spectrum of compound 16 in Pyridine-d5. | 17 |
| 30 | **Fig S30.** IR spectrum of compound 16. | 17 |
| 31 | **Fig S31.** HR-ESI-MS spectrum of compound 16. | 18 |
| 32 | **Fig S32.** UV-Vis spectrum of compound 16 in CH3OH. | 18 |


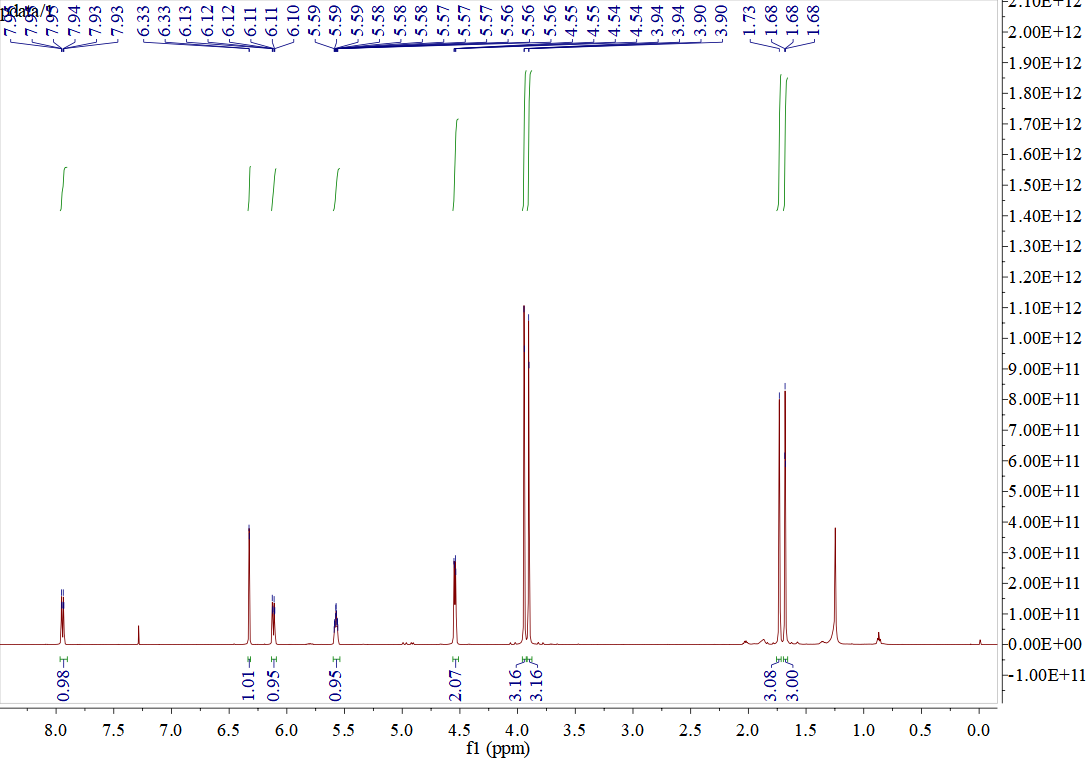

**Fig S1** ^1^H-NMR spectrum of compound **4** in CDCl_3_ (600 MHz).

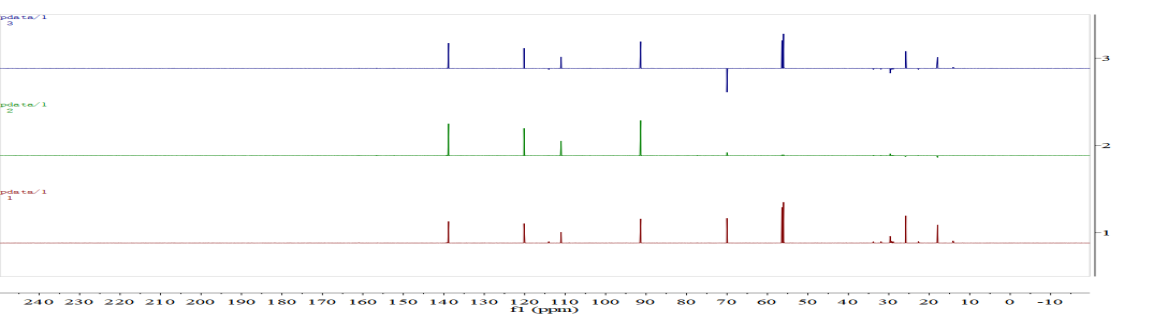


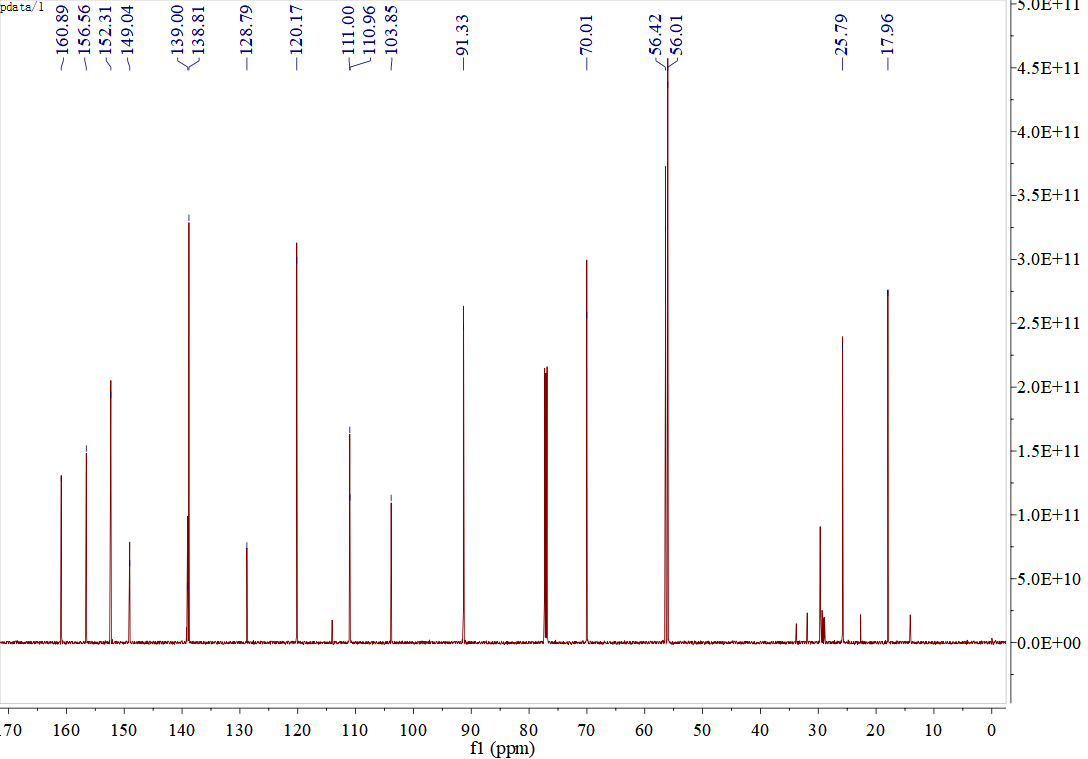


**Fig S2** ^13^C-NMR spectrum and DEPT of compound **4** in CDCl_3_ (151 MHz).


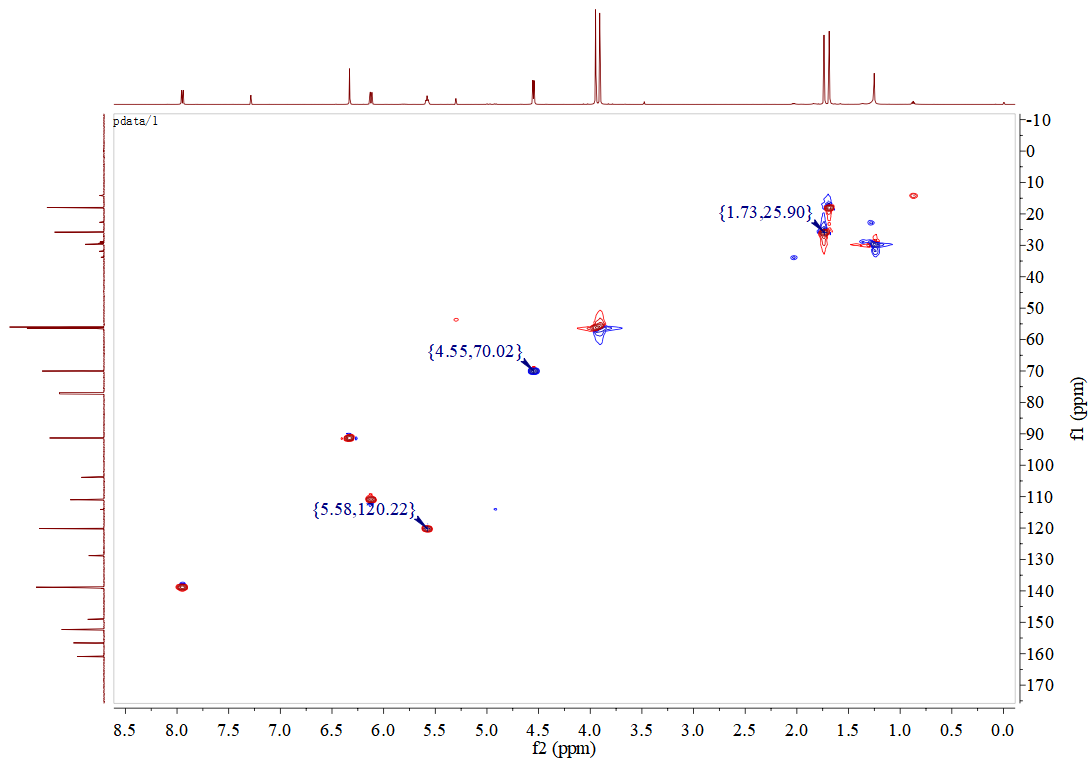

**Fig S3** HSQC spectrum of compound **4** in CDCl_3_.


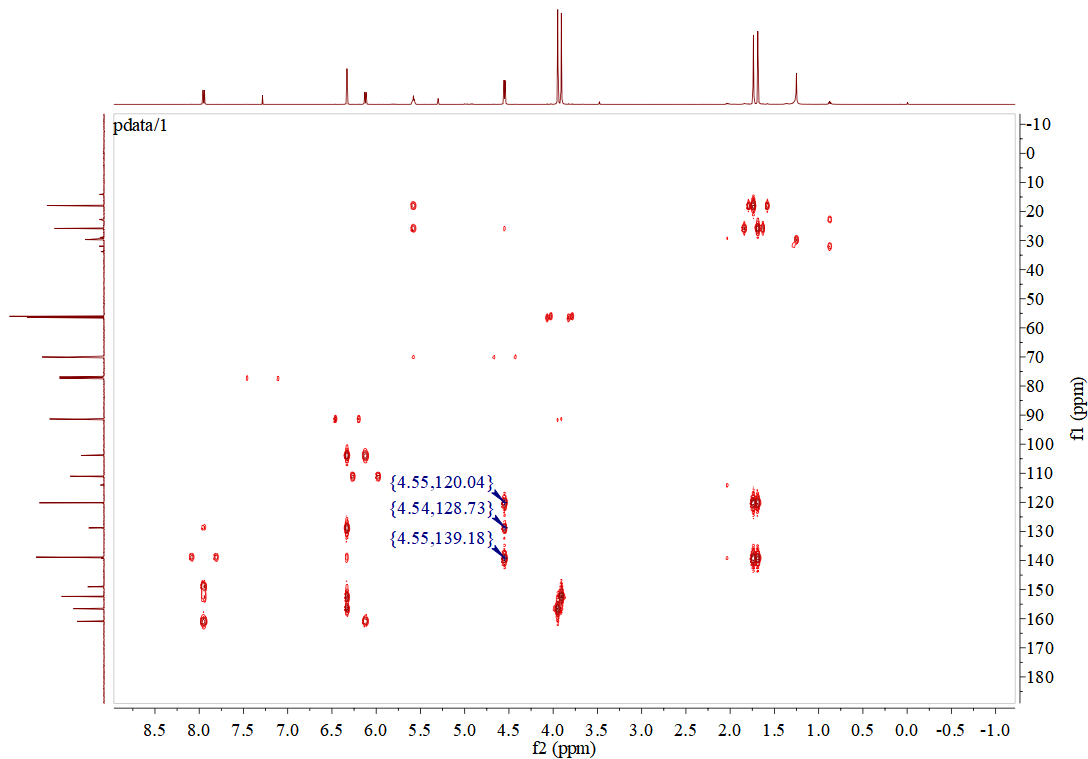

**Fig S4** HMBC spectrum of compound **4** in CDCl_3_.


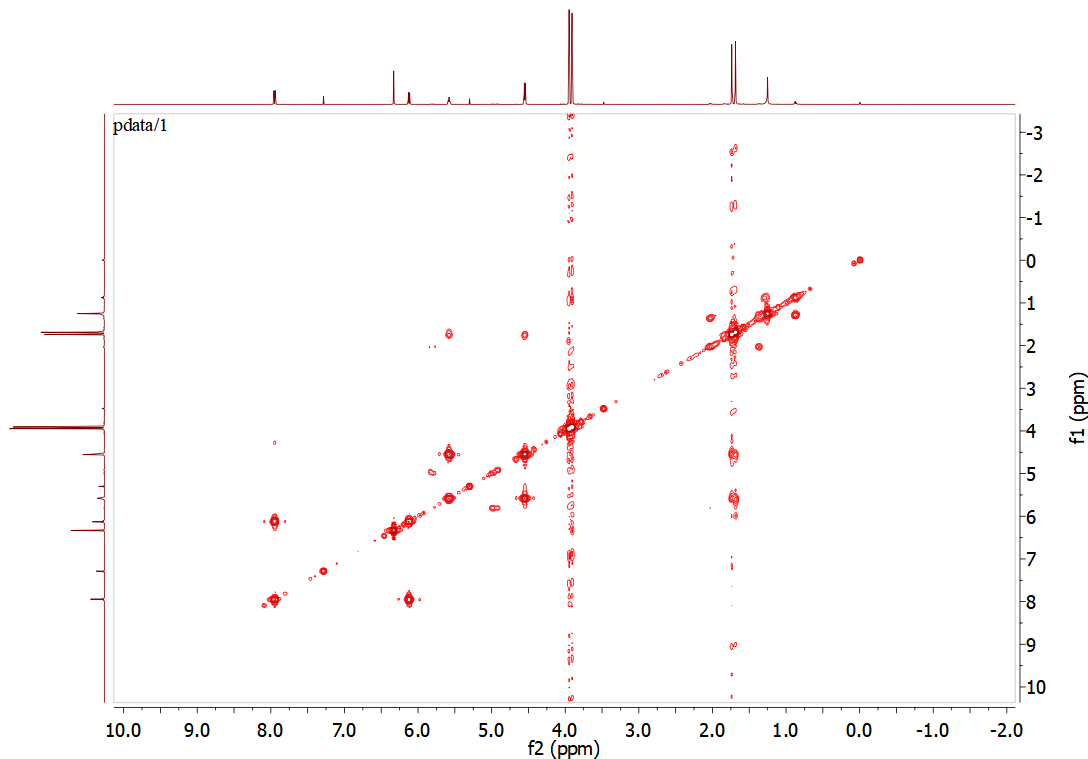

**Fig S5** ^1^H- ^1^H COSY spectrum of compound **4** in in CDCl_3_.


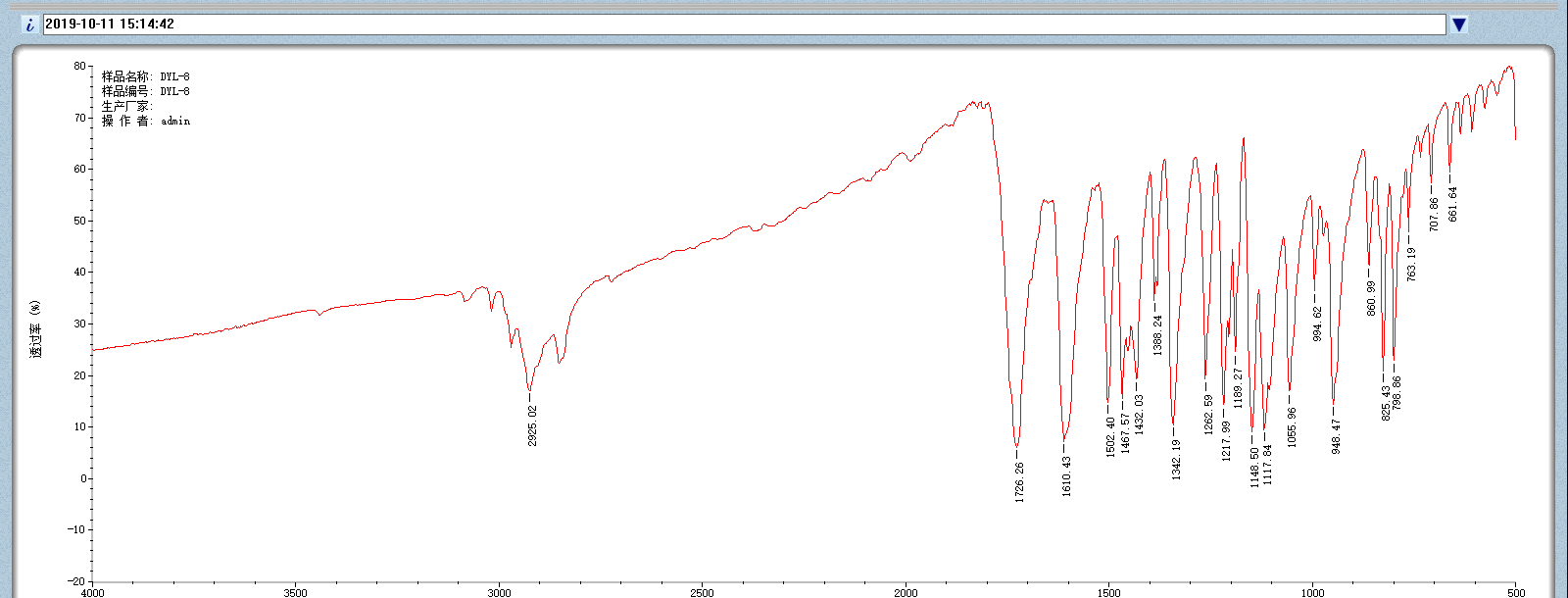


**Fig S6** IR spectrum of compound **4**.

**Fig S7** HR-ESI-MS spectrum of compound **4**.





**Fig S8** UV-Vis spectrum of compound **4** in CH_3_OH.


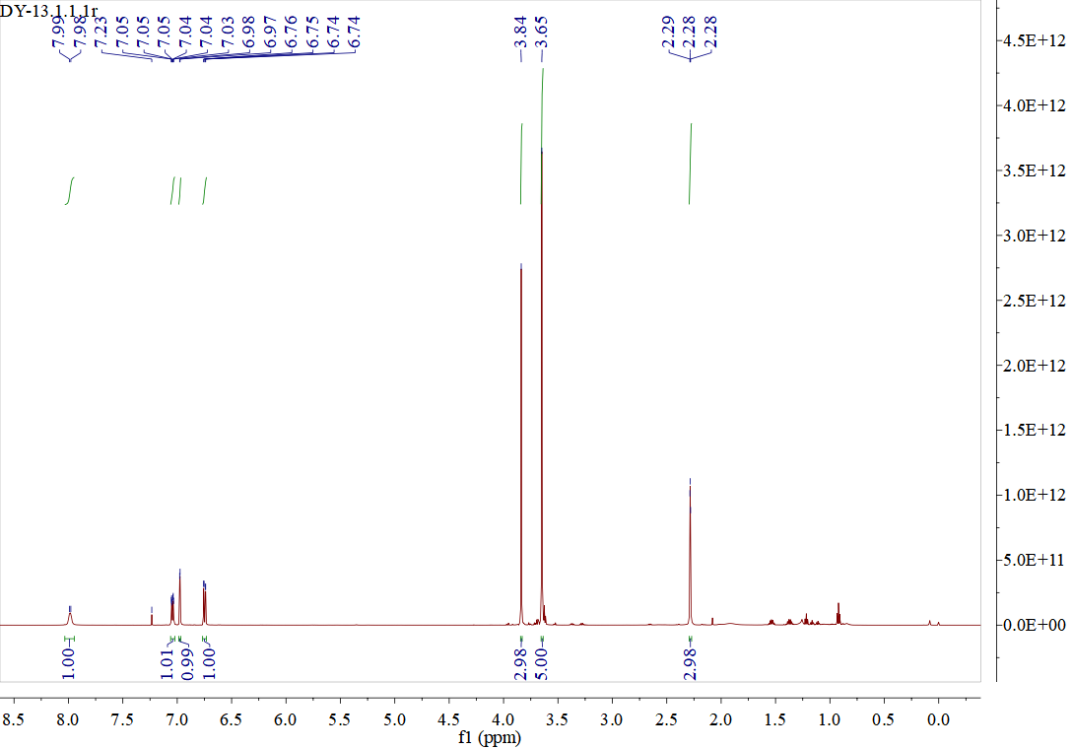

**Fig S9** ^1^H-NMR spectrum of compound **5** in CDCl_3_ (600 MHz).

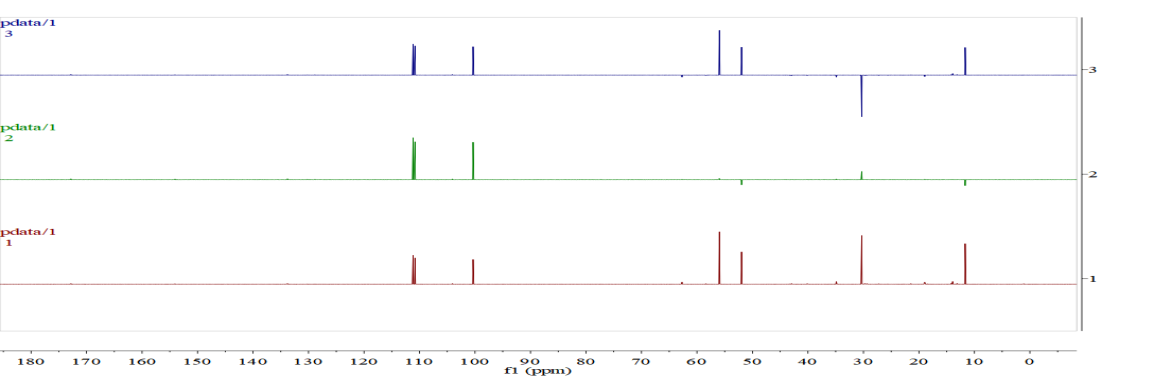

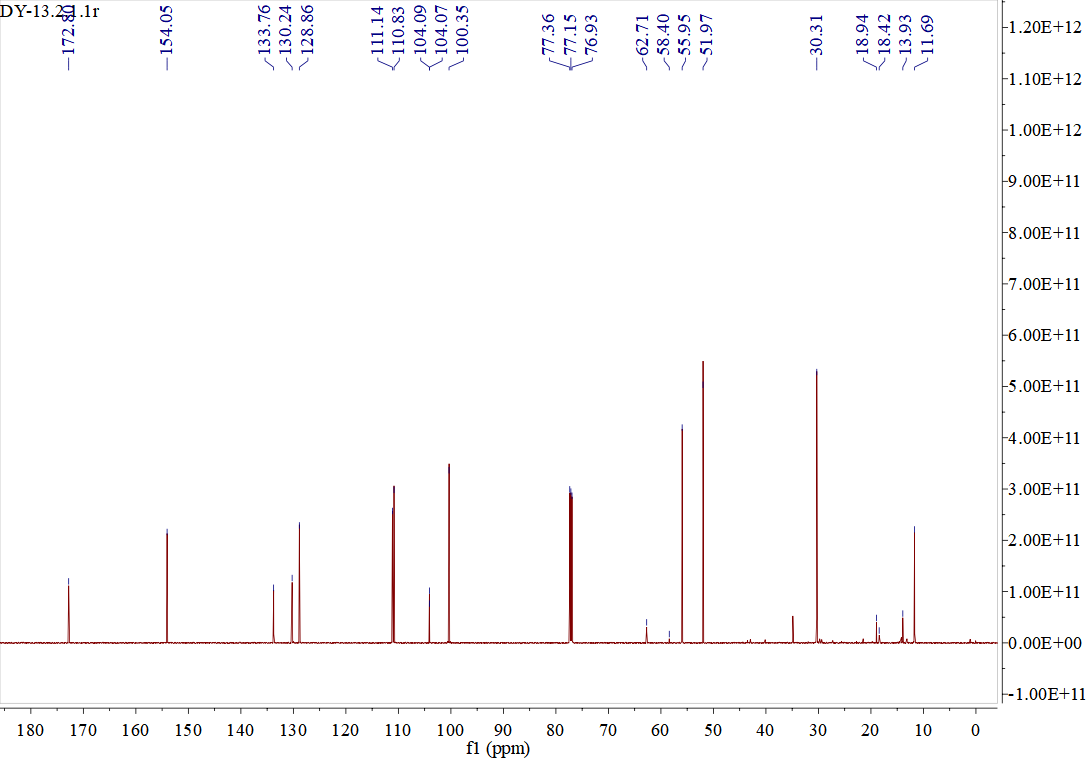


**Fig S10** ^13^C-NMR spectrum and DEPT of compound **5** in CDCl_3_ (151 MHz).


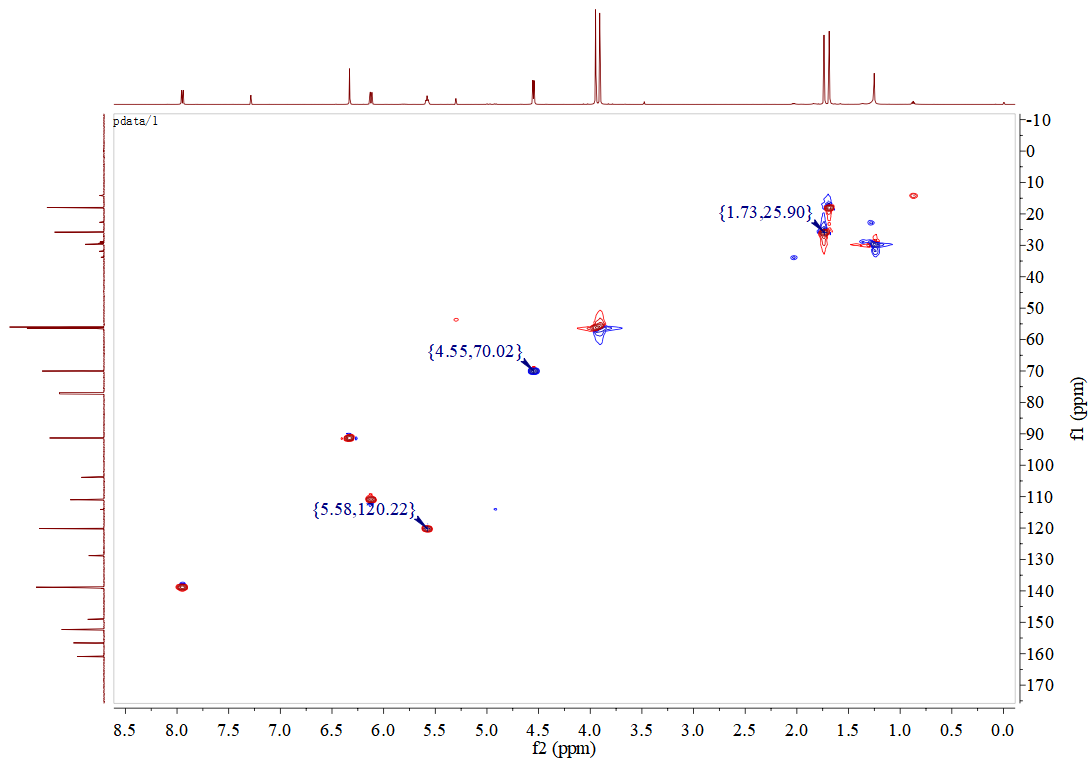

**Fig S11** HSQC spectrum of compound **5** in CDCl_3_.


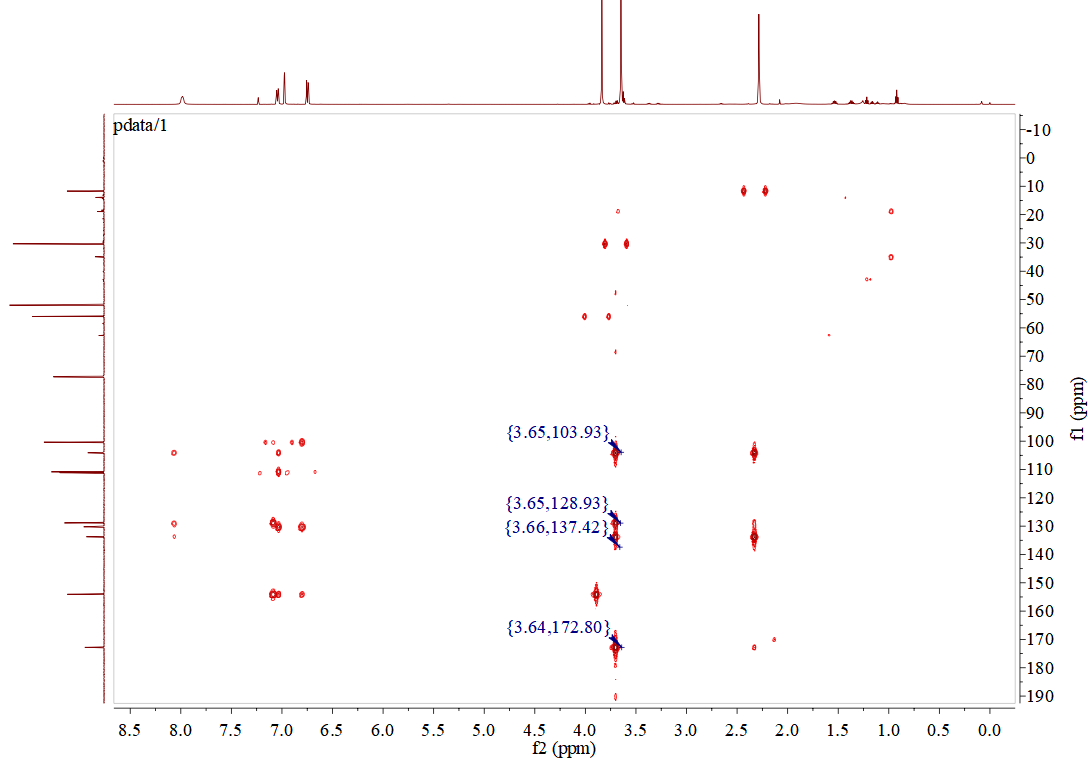

**Fig S12** HMBC spectrum of compound **5** in CDCl_3_.


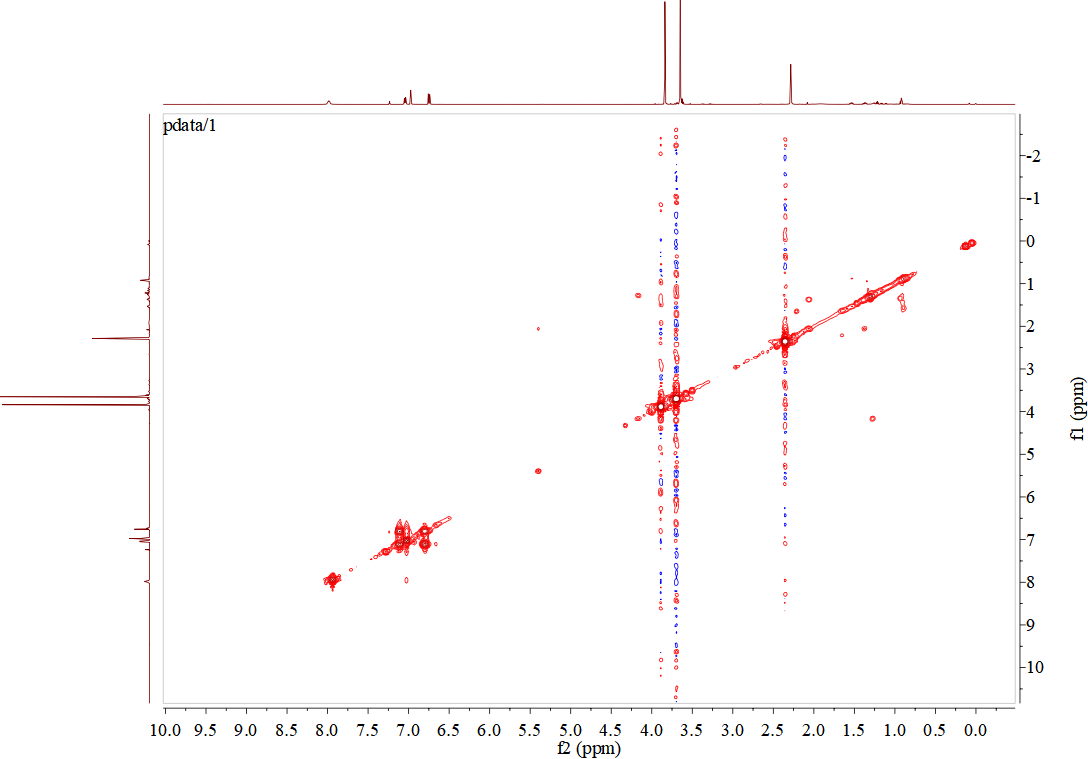

**Fig S13** ^1^H- ^1^H COSY spectrum of compound **5** in CDCl_3_.


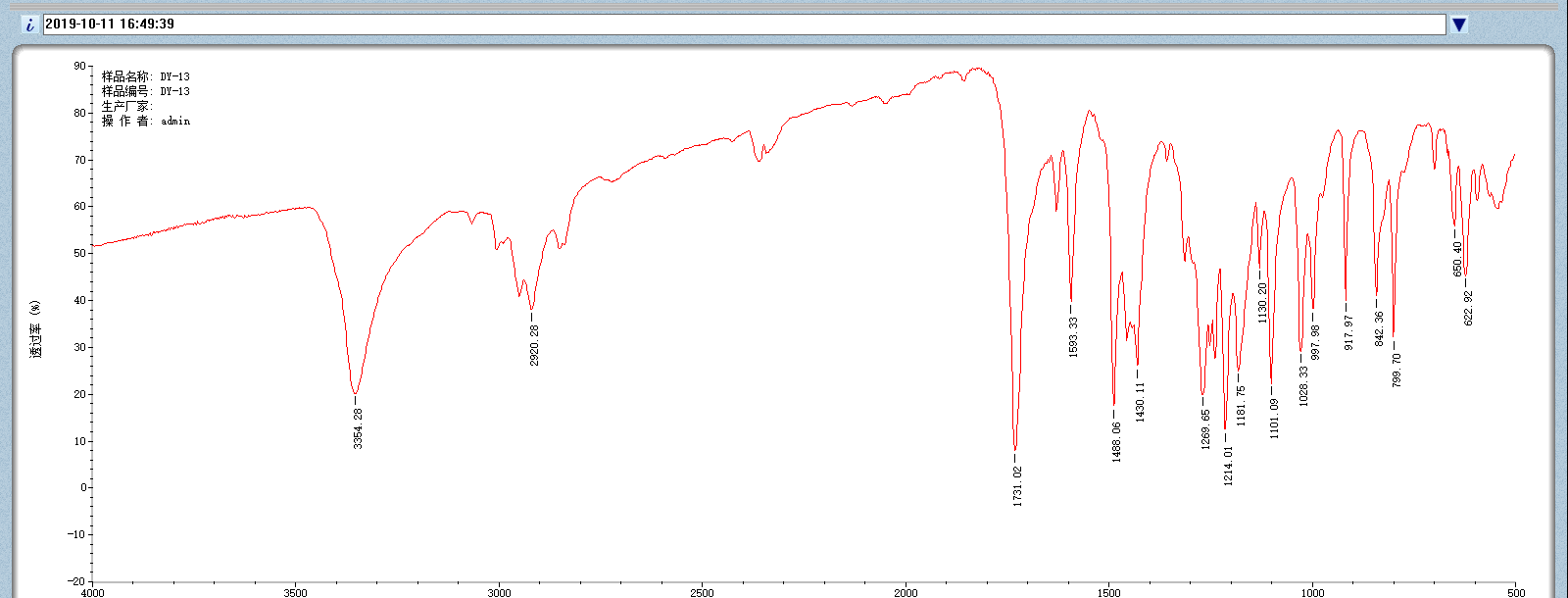


**Fig S14** IR spectrum of compound **5**.

**Fig S15** HR-ESI-MS spectrum of compound **5**.





**Fig S16** UV-Vis spectrum of compound **5** in CH_3_OH.


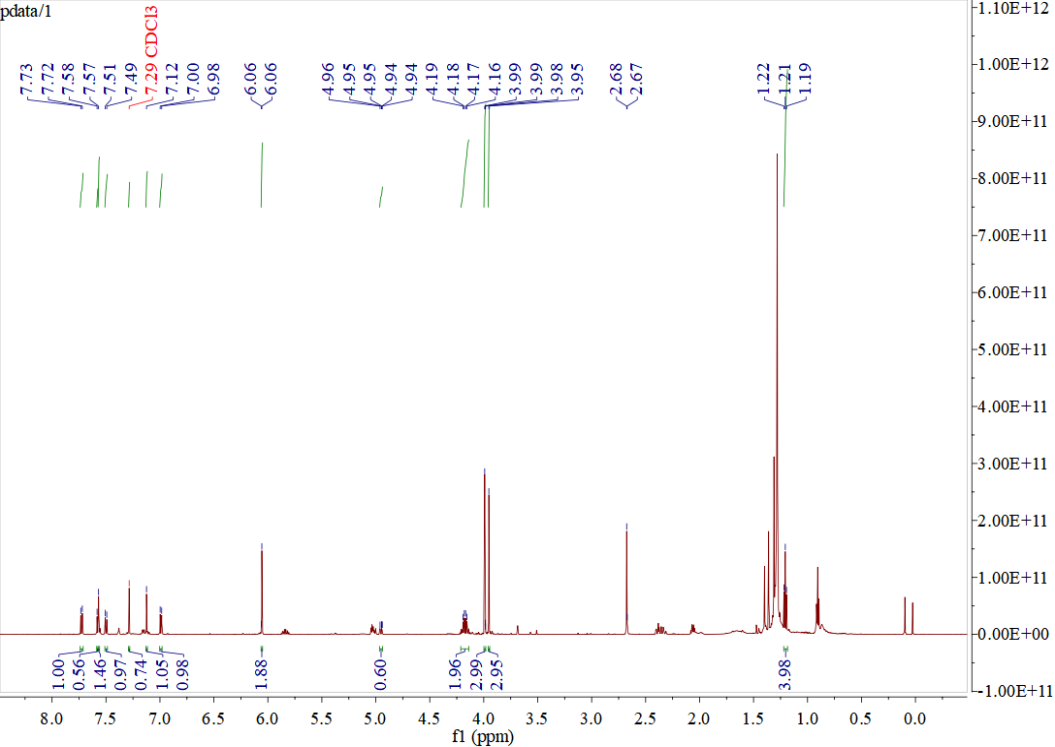

**Fig S17** ^1^H-NMR spectrum of compound **6** in CDCl_3_ (600 MHz).


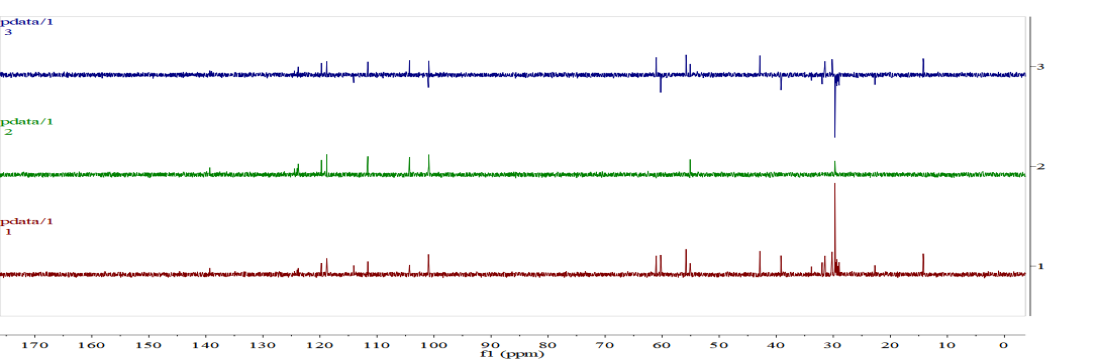


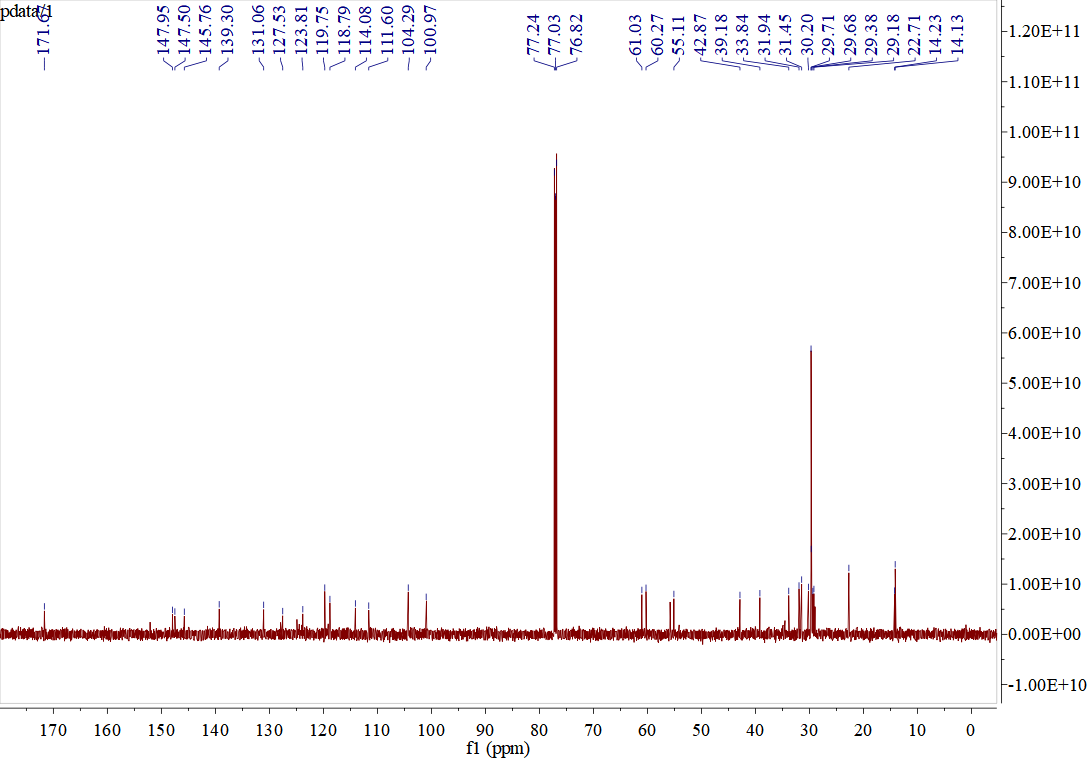

**Fig S18** ^13^C-NMR spectrum and DEPT of compound **6** in CDCl_3_ (151 MHz).


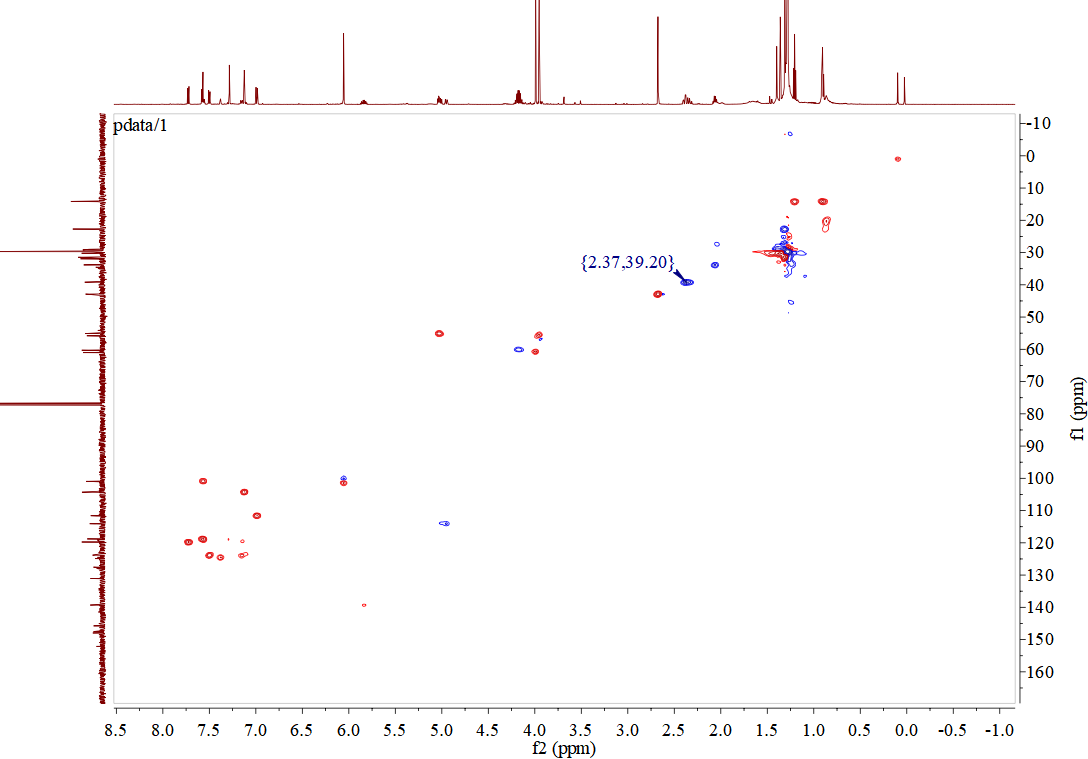

**Fig S19** HSQC spectrum of compound **6** in CDCl_3_.


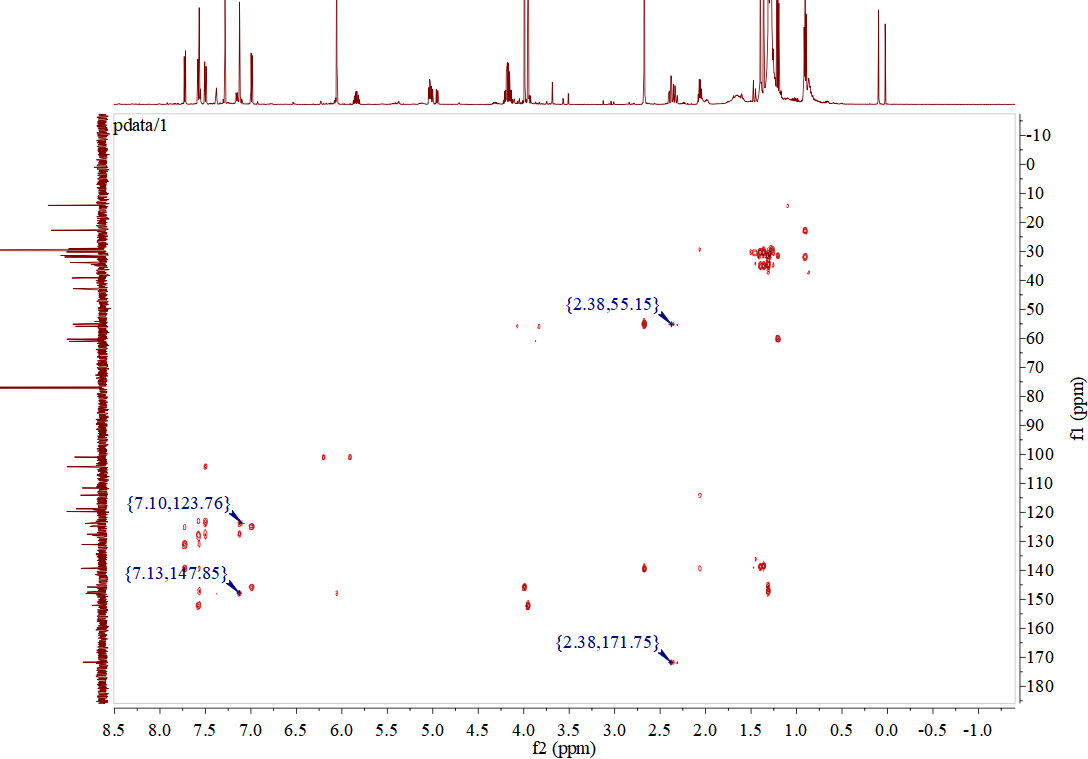

**Fig S20** HMBC spectrum of compound **6** in CDCl_3_.


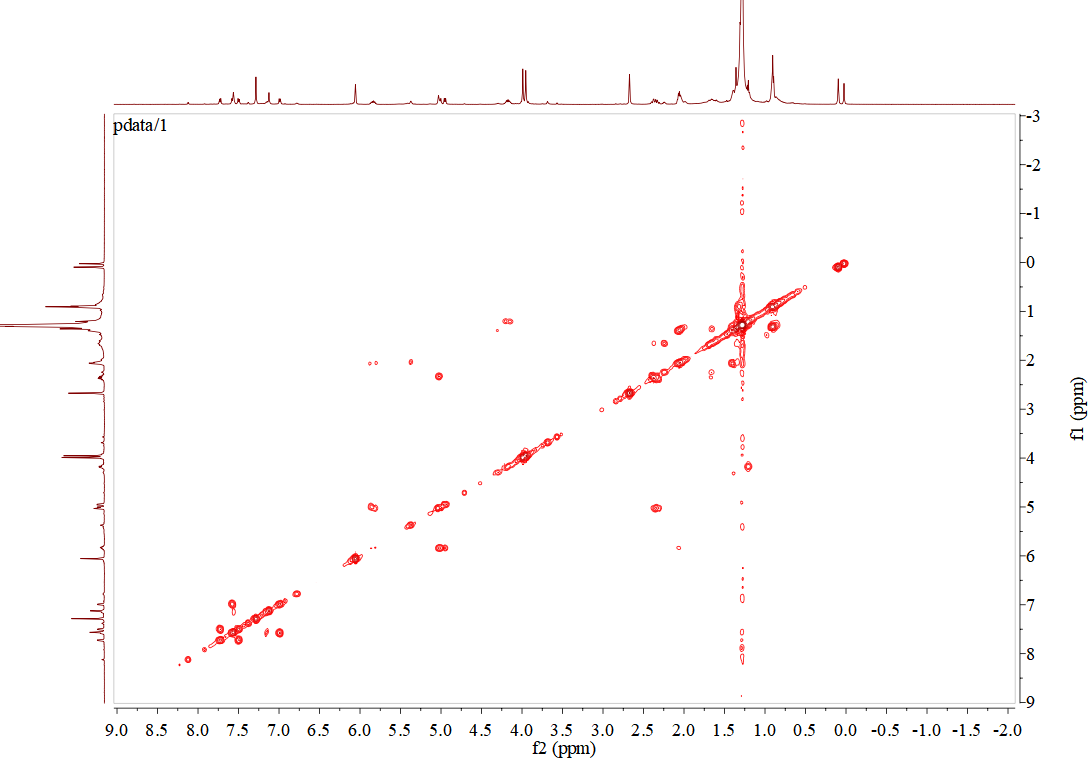

**Fig S21** ^1^H- ^1^H COSY spectrum of compound **6** in CDCl_3_.


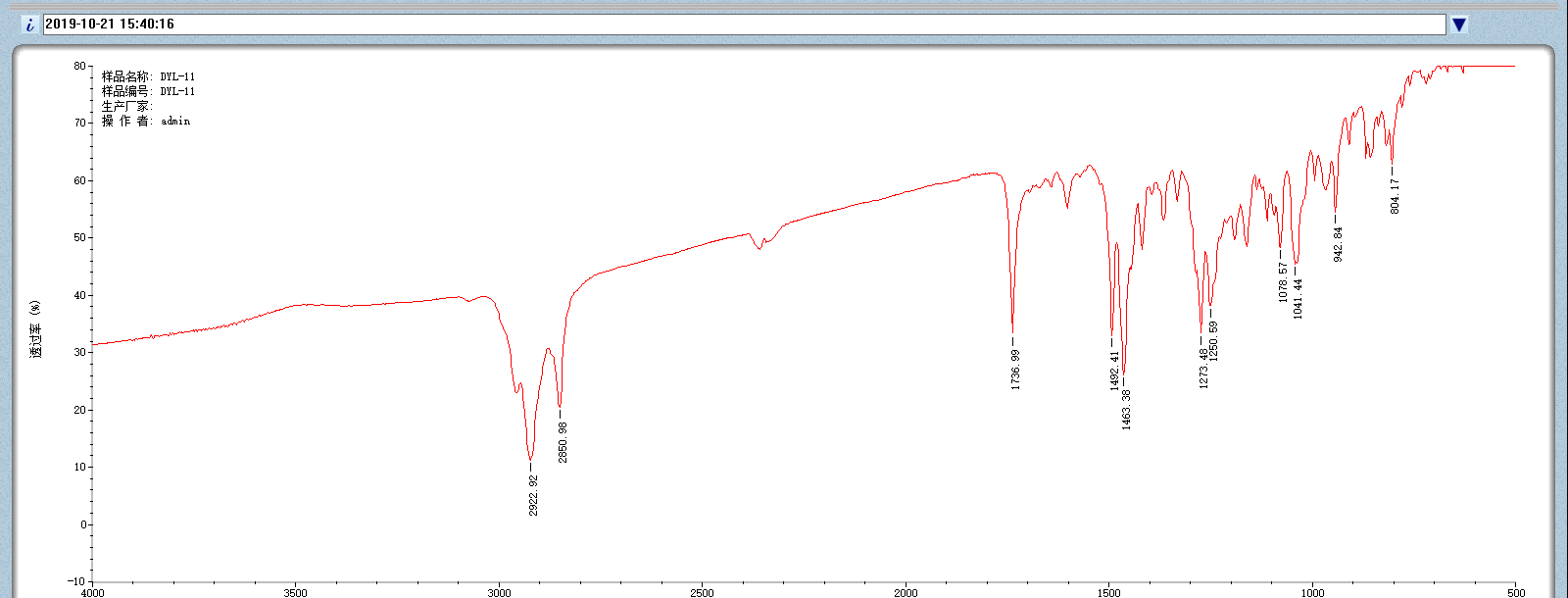


**Fig S22** IR spectrum of compound **6**.

**Fig S23** HR-ESI-MS spectrum of compound **6**.





**Fig S24** UV-Vis spectrum of compound **6** in CH_3_OH.


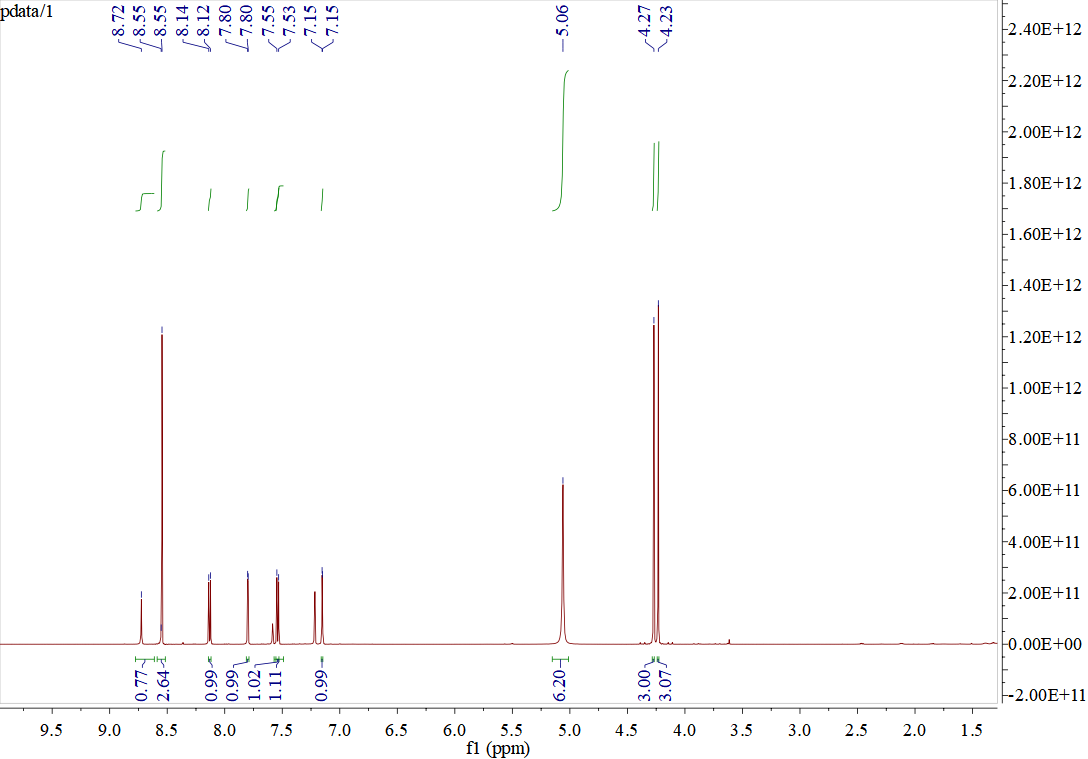

**Fig S25** ^1^H-NMR spectrum of compound **16** in Pyridine-*d*_5_ (600 MHz).


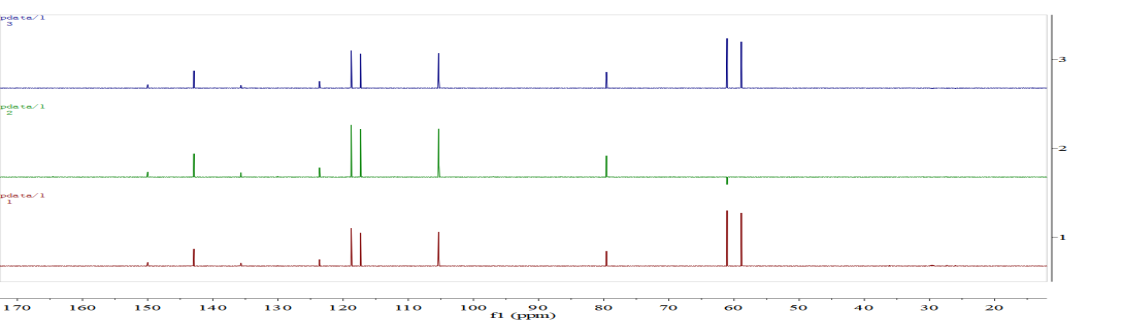


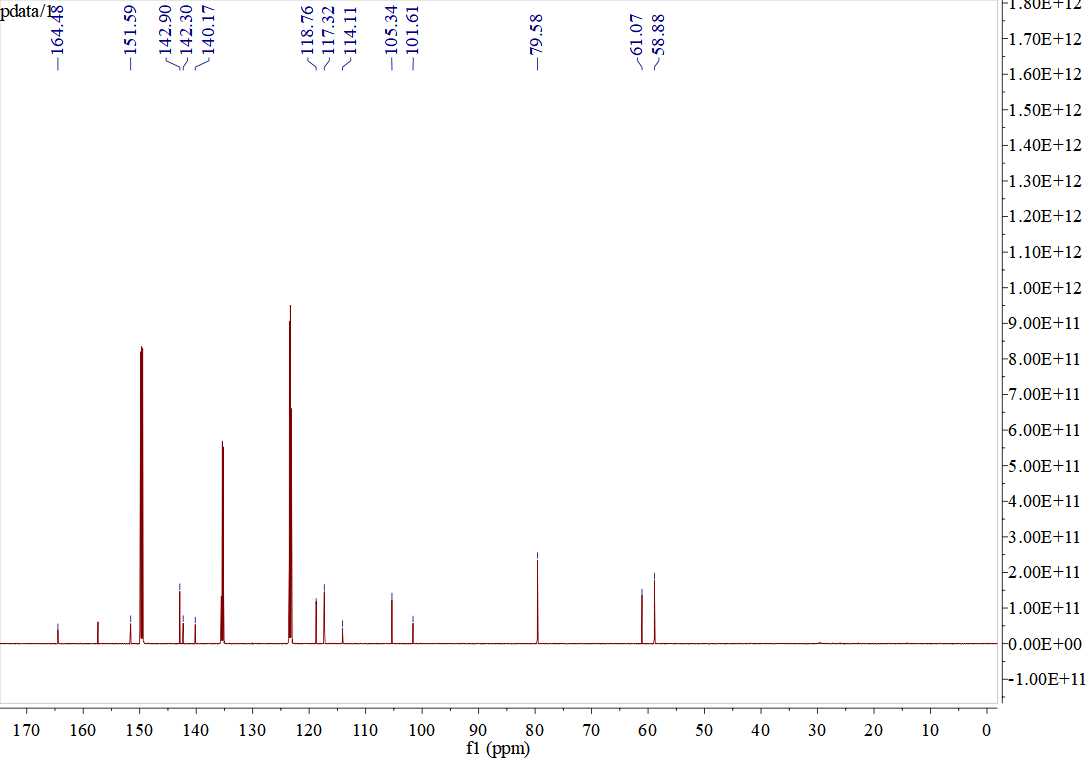

**Fig S26** ^13^C-NMR spectrum and DEPT of compound **16** in Pyridine-*d*_5_ (151 MHz).


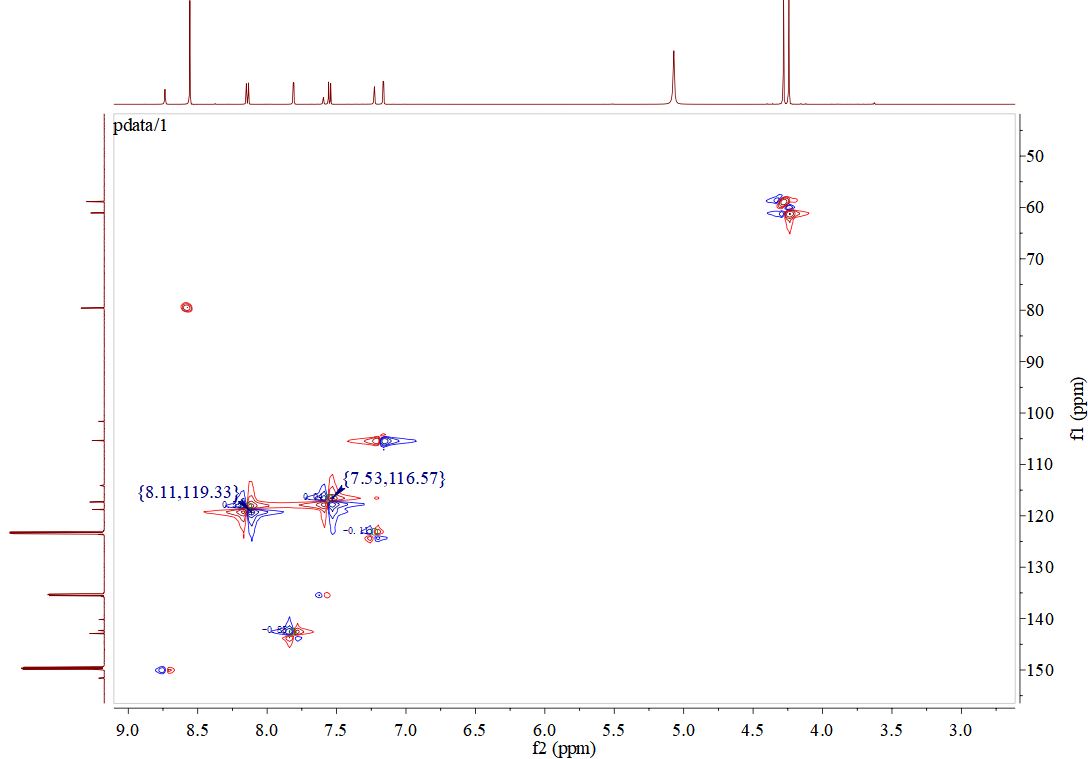

**Fig S27** HSQC spectrum of compound **16** in Pyridine-*d*_5_


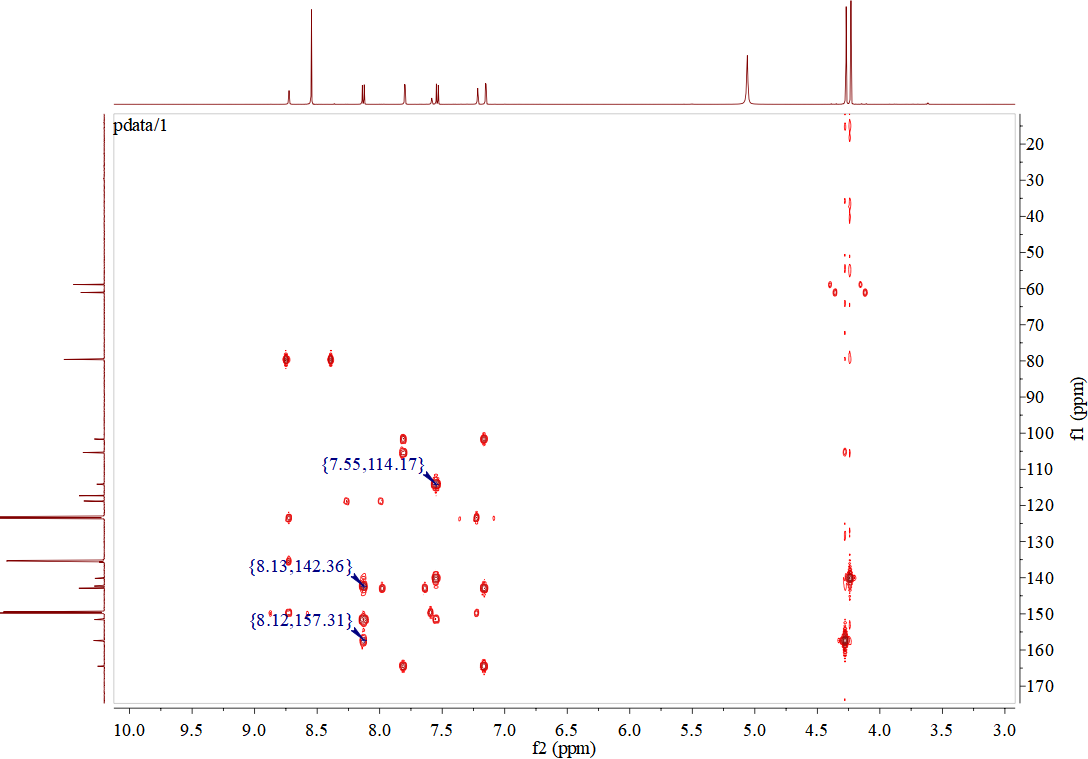

**Fig S28** HMBC spectrum of compound **16** in Pyridine-*d*_5_.


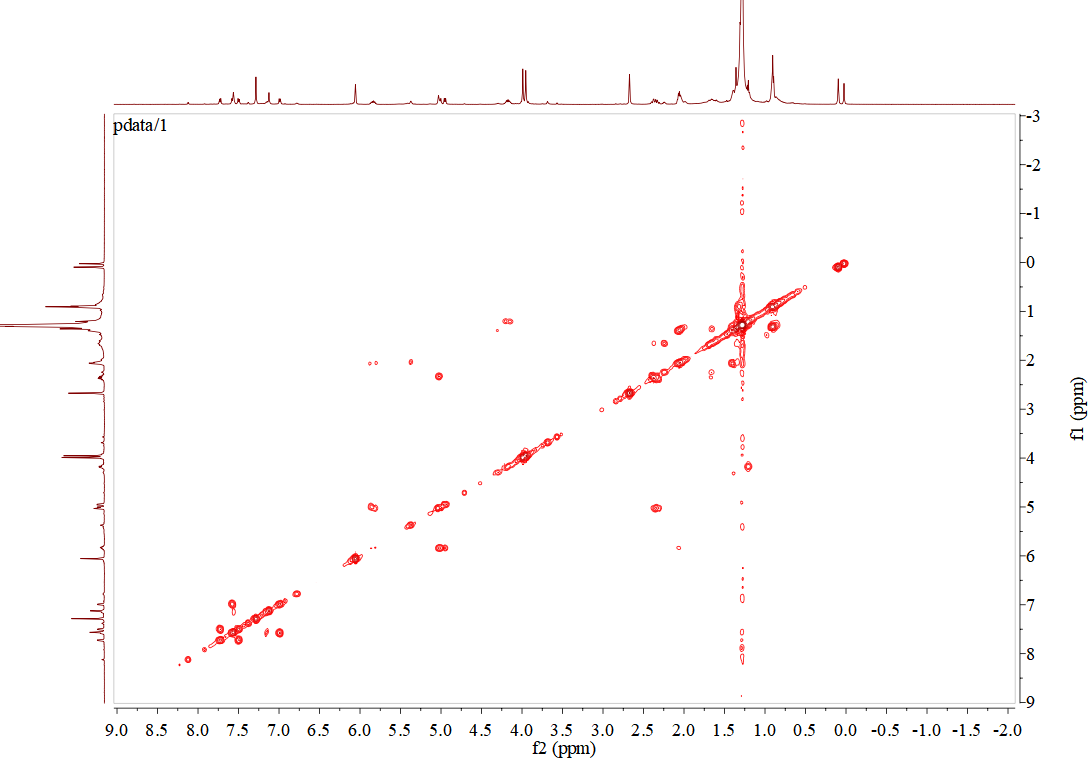

**Fig S29** ^1^H- ^1^H COSY spectrum of compound **16** in Pyridine-*d*_5_.


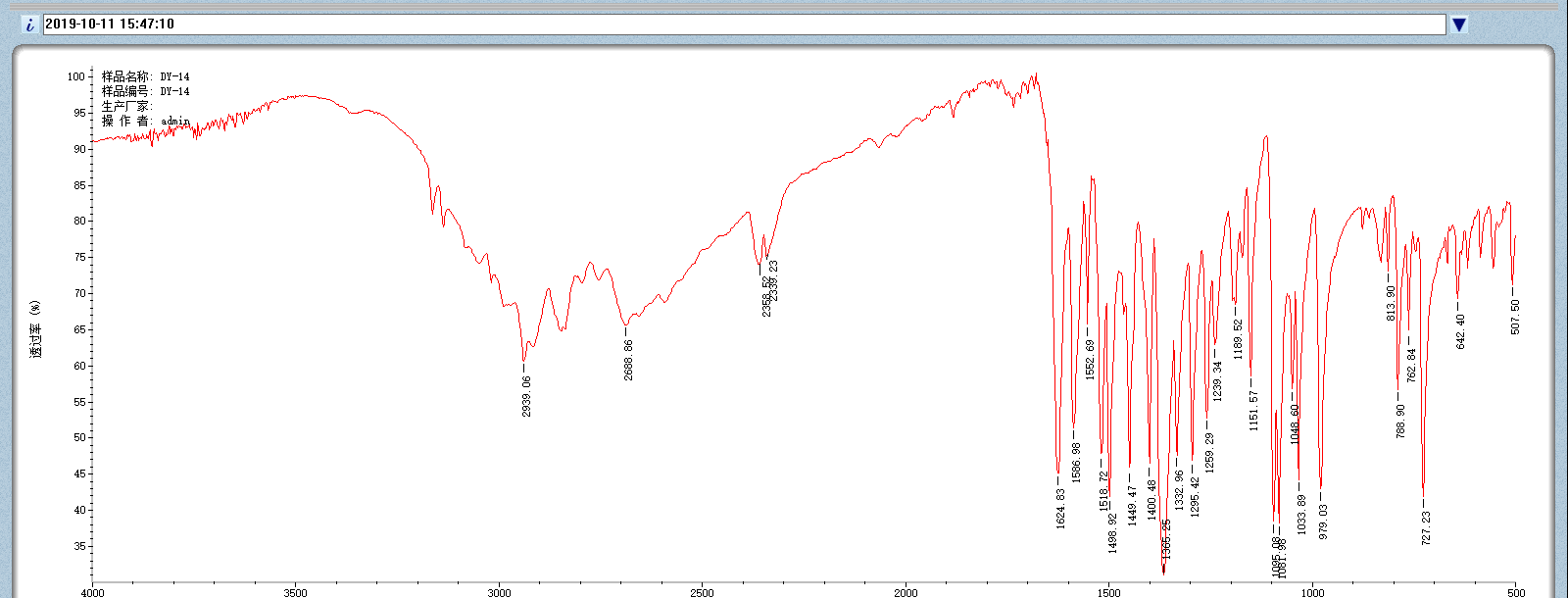


**Fig S30** IR spectrum of compound **16**.

**Fig S31** HR-ESI-MS spectrum of compound **16**.





**Fig S32** UV-Vis spectrum of compound **16** in CH_3_OH.

**Supporting Information List of Contents 2**

| **No.** | **Content** | **Page** |
| --- | --- | --- |
| 1 | **Fig SS1.** 1H-NMR spectrum of compound **1** in CDCl3. | 20 |
| 2 | **Fig SS2.** ESI-MS spectrum of compound 1. | 20 |
| 3 | **Fig SS3.** 1H-NMR spectrum of compound **2** in CDCl3. | 21 |
| 4 | **Fig SS4.** ESI-MS spectrum of compound 2. | 21 |
| 5 | **Fig SS5.** 1H-NMR spectrum of compound **3** in CDCl3. | 22 |
| 6 | **Fig SS6**. ESI-MS spectrum of compound 3. | 22 |
| 7 | **Fig SS7.** 1H-NMR spectrum of compound **7** in CDCl3. | 23 |
| 8 | **Fig SS8.** ESI-MS spectrum of compound 7. | 23 |
| 9 | **Fig SS9.** 1H-NMR spectrum of compound **8** in CDCl3. | 24 |
| 10 | **Fig SS10.** ESI-MS spectrum of compound 8. | 24 |
| 13 | **Fig SS11.** 1H-NMR spectrum of compound **10** in CDCl3. | 25 |
| 14 | **Fig SS12.** ESI-MS spectrum of compound 10. | 25 |
| 15 | **Fig SS13.** 1H-NMR spectrum of compound **11** in CDCl3. | 26 |
| 16 | **Fig SS14.** ESI-MS spectrum of compound 11. | 26 |
| 17 | **Fig SS15.** 1H-NMR spectrum of compound **12** in CDCl3. | 27 |
| 18 | **Fig SS16.** ESI-MS spectrum of compound 12. | 27 |
| 19 | **Fig SS17.** 1H-NMR spectrum of compound **13** in CDCl3. | 28 |
| 20 | **Fig SS18.** ESI-MS spectrum of compound 13. | 28 |
| 23 | **Fig SS19.** 1H-NMR spectrum of compound **15** in CDCl3. | 29 |
| 24 | **Fig SS20.** ESI-MS spectrum of compound 15. | 29 |
| 25 | **Fig SS21.** 1H-NMR spectrum of compound **17** in CDCl3. | 30 |
| 26 | **Fig SS22.** ESI-MS spectrum of compound 17. | 30 |
| 29 | **Fig SS23.** 1H-NMR spectrum of compound **19** in CDCl3. | 31 |
| 30 | **Fig SS24.** ESI-MS spectrum of compound 19. | 31 |
| 39 | **Fig SS25.** 1H-NMR spectrum of compound **24** in CDCl3. | 32 |
| 40 | **Fig SS26.** ESI-MS spectrum of compound 24. | 32 |


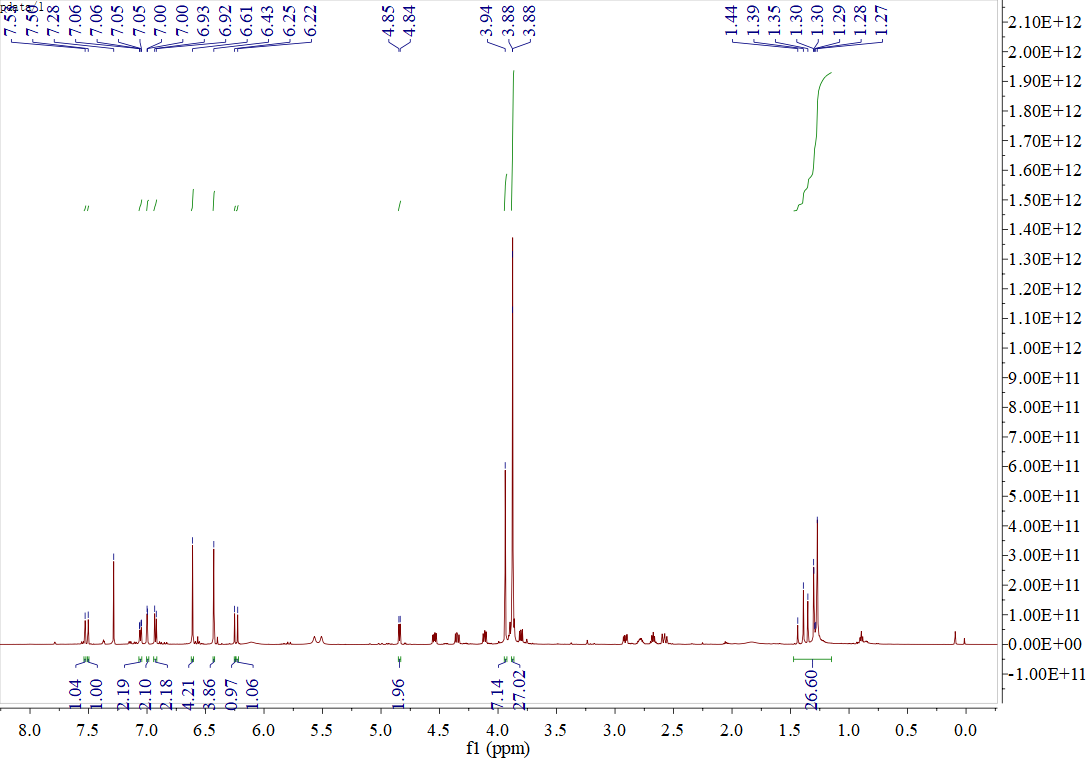


**Fig SS1.** ^1^H-NMR spectrum of compound **1** in CDCl_3_ (600 MHz).


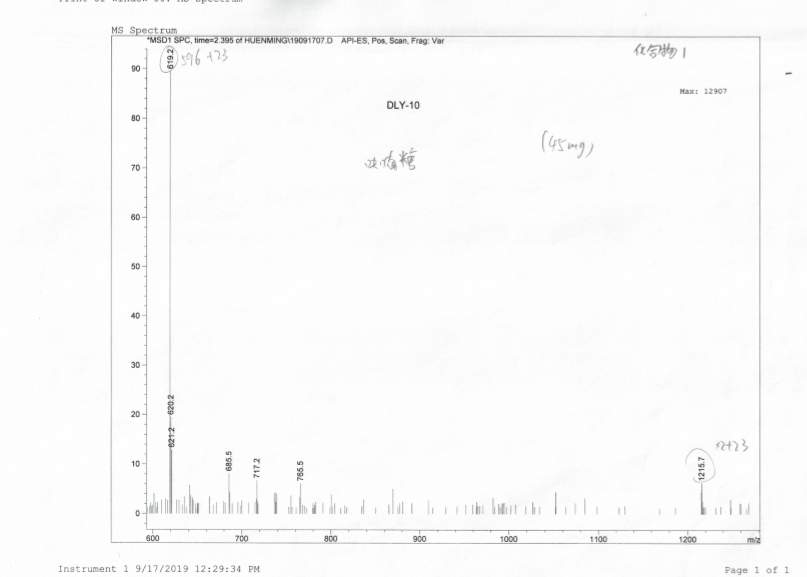


**Fig SS2.** ESI-MS spectrum of compound **1**.


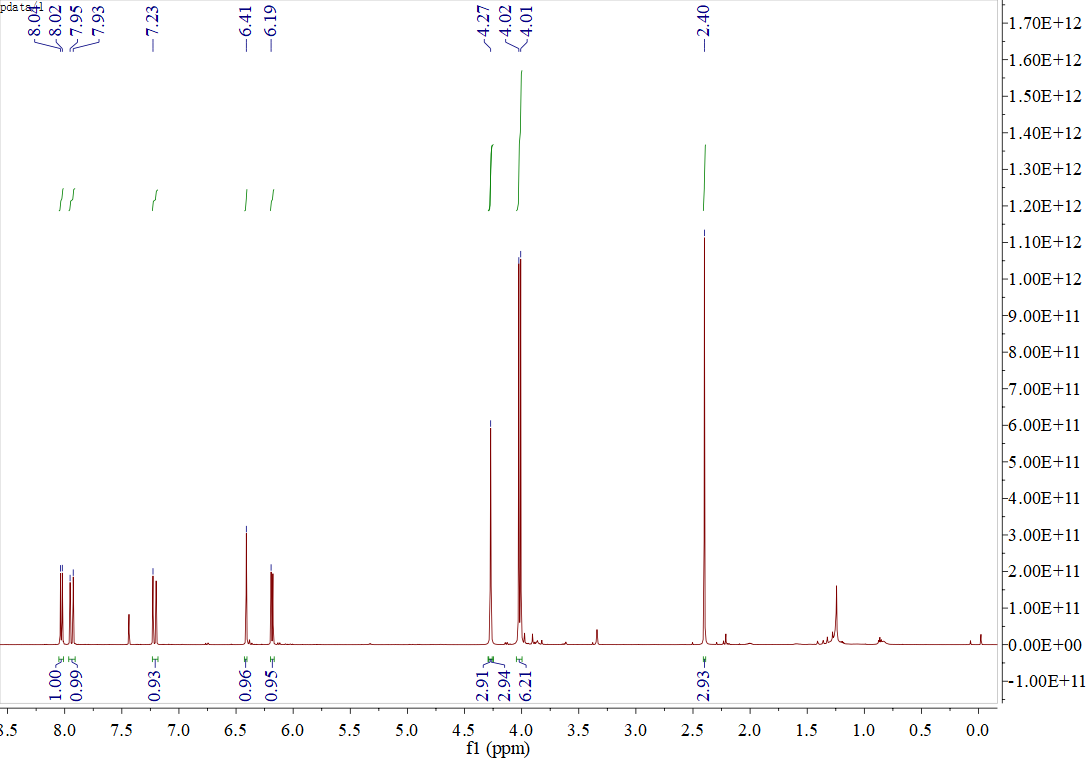


**Fig SS3.** ^1^H-NMR spectrum of compound **2** in CDCl_3_ (600 MHz).


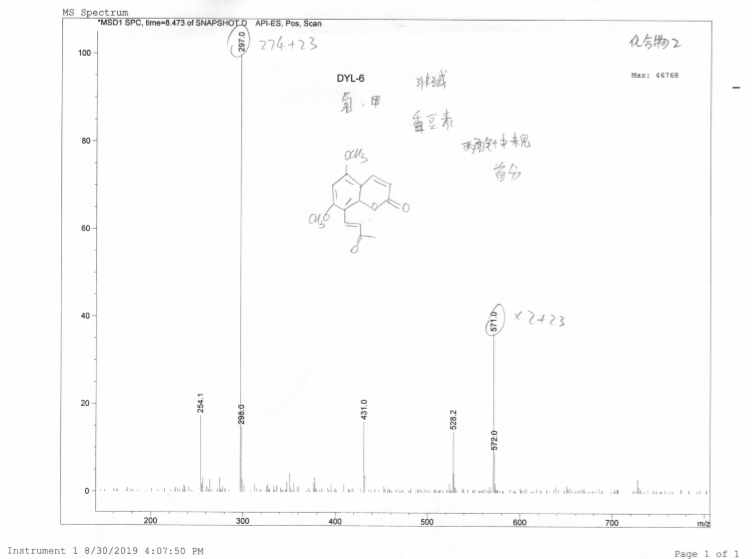


**Fig SS4.** ESI-MS spectrum of compound **2**.


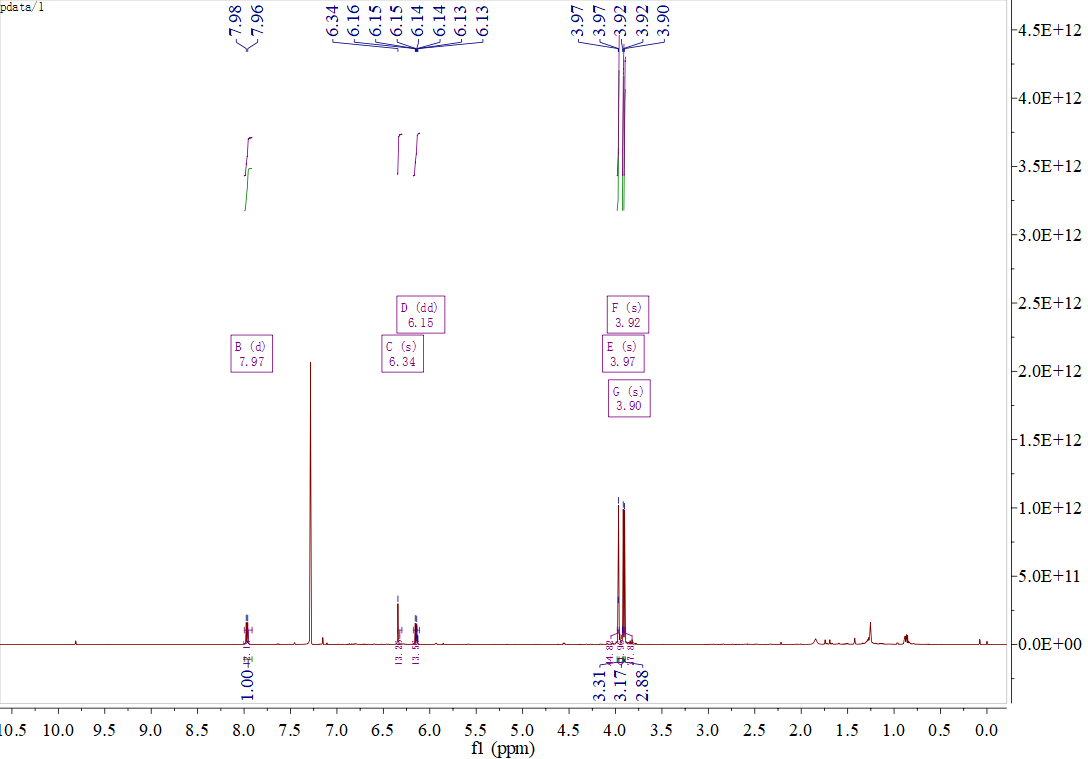


**Fig SS5.** ^1^H-NMR spectrum of compound **3** in CDCl_3_ (600 MHz).


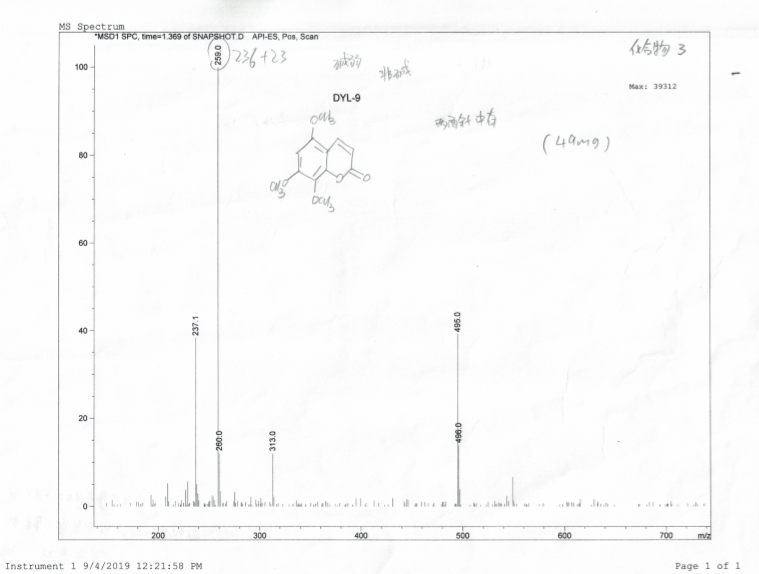


**Fig SS6.** ESI-MS spectrum of compound **3**.


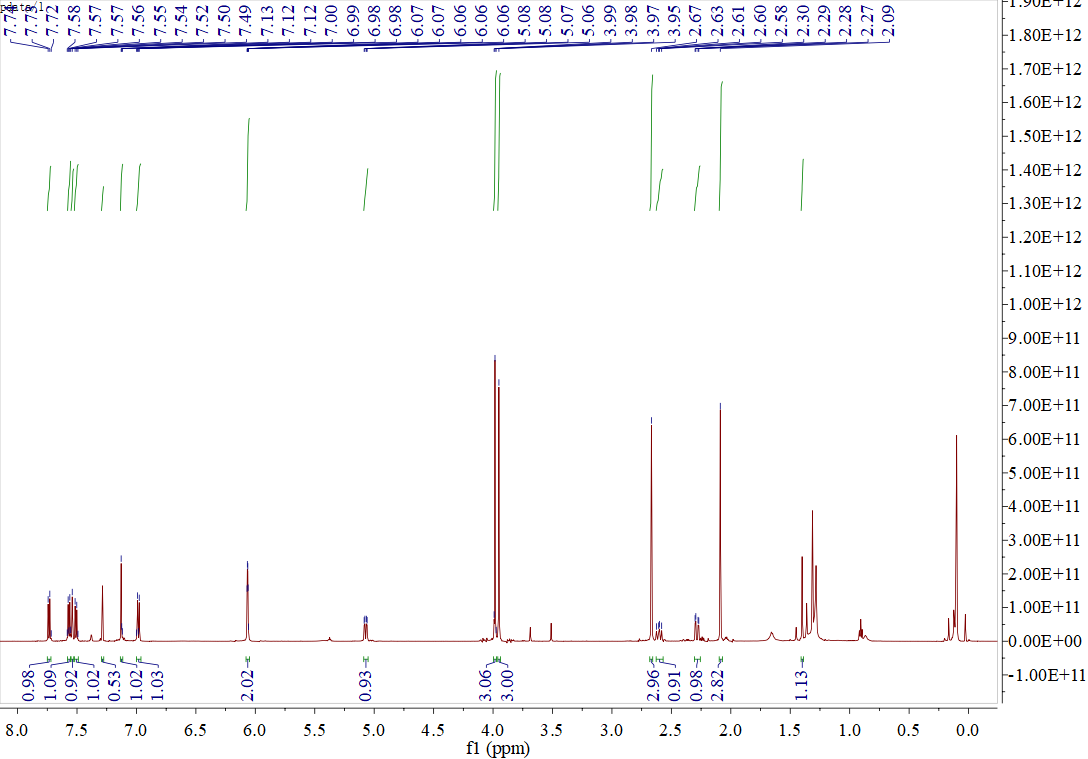


**Fig SS7** ^1^H-NMR spectrum of compound **7** in CDCl_3_ (600 MHz).


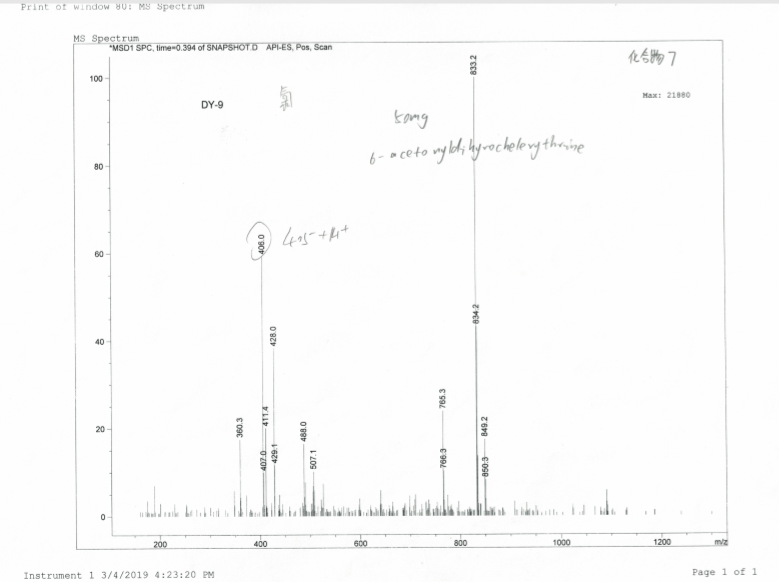


**Fig SS8.** ESI-MS spectrum of compound **7**.


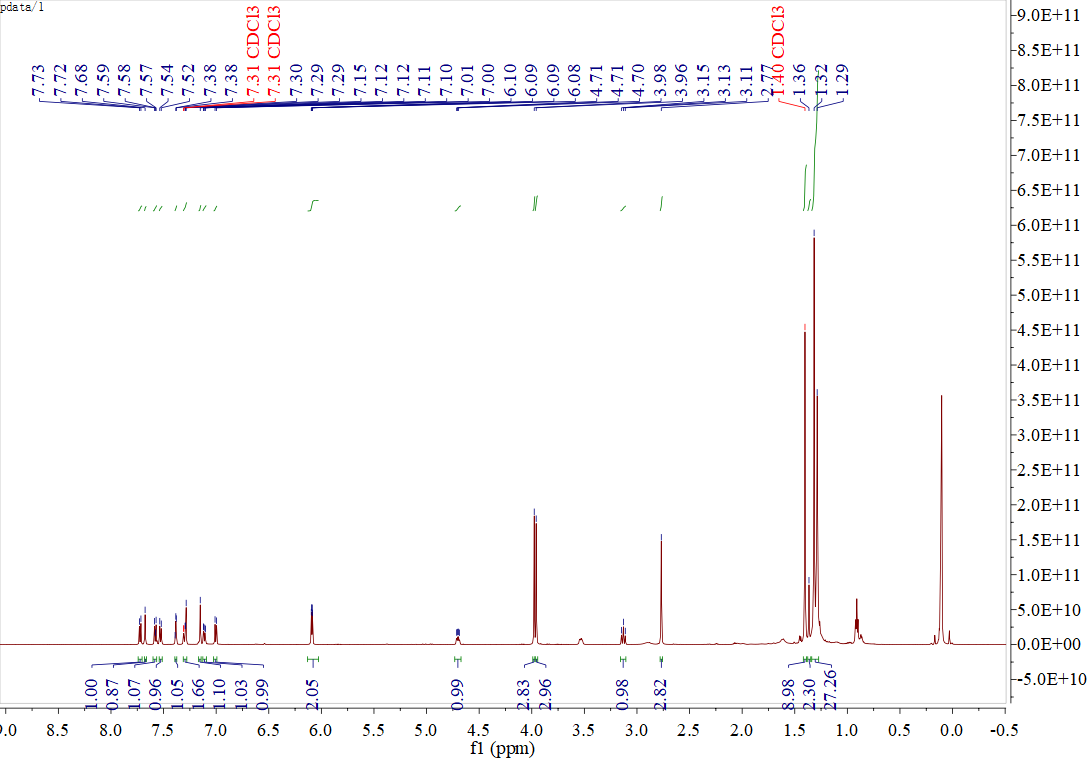


**Fig SS9.** ^1^H-NMR spectrum of compound **8** in CDCl_3_ (600 MHz).


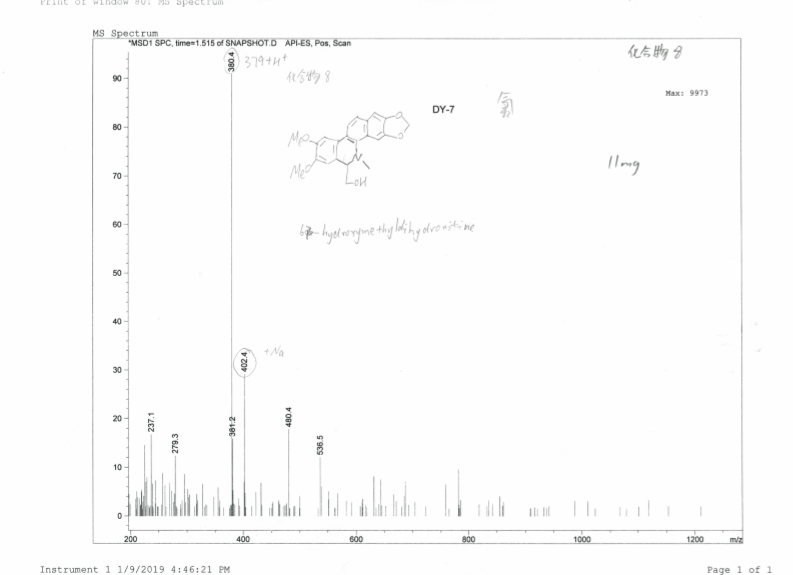


**Fig SS10.** ESI-MS spectrum of compound **8**.


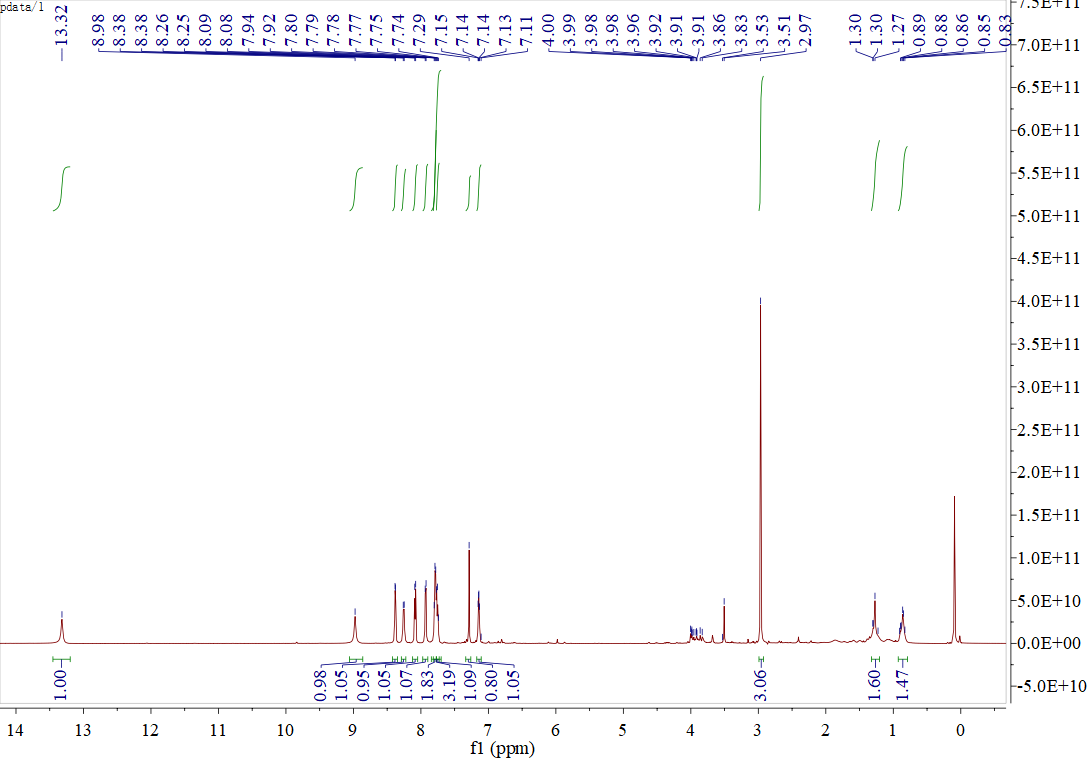


**Fig SS11.** ^1^H-NMR spectrum of compound **10** in CDCl_3_ (600 MHz).


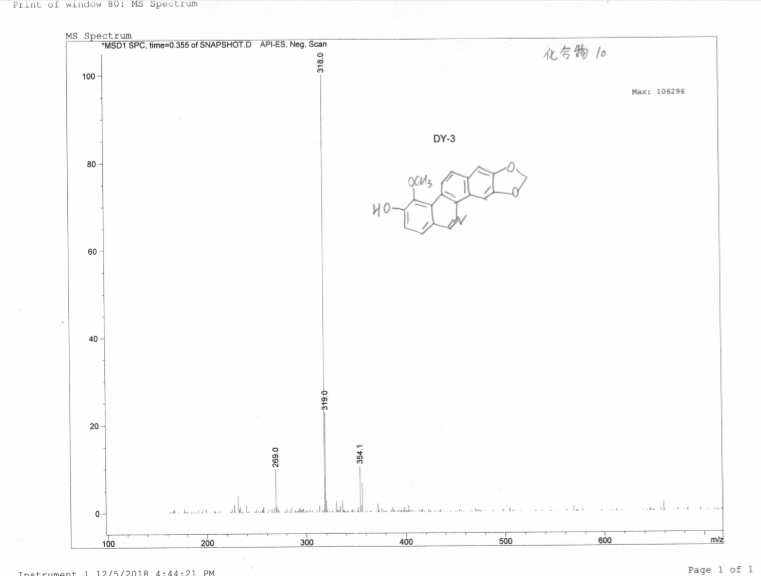


**Fig SS12.** ESI-MS spectrum of compound **10**.


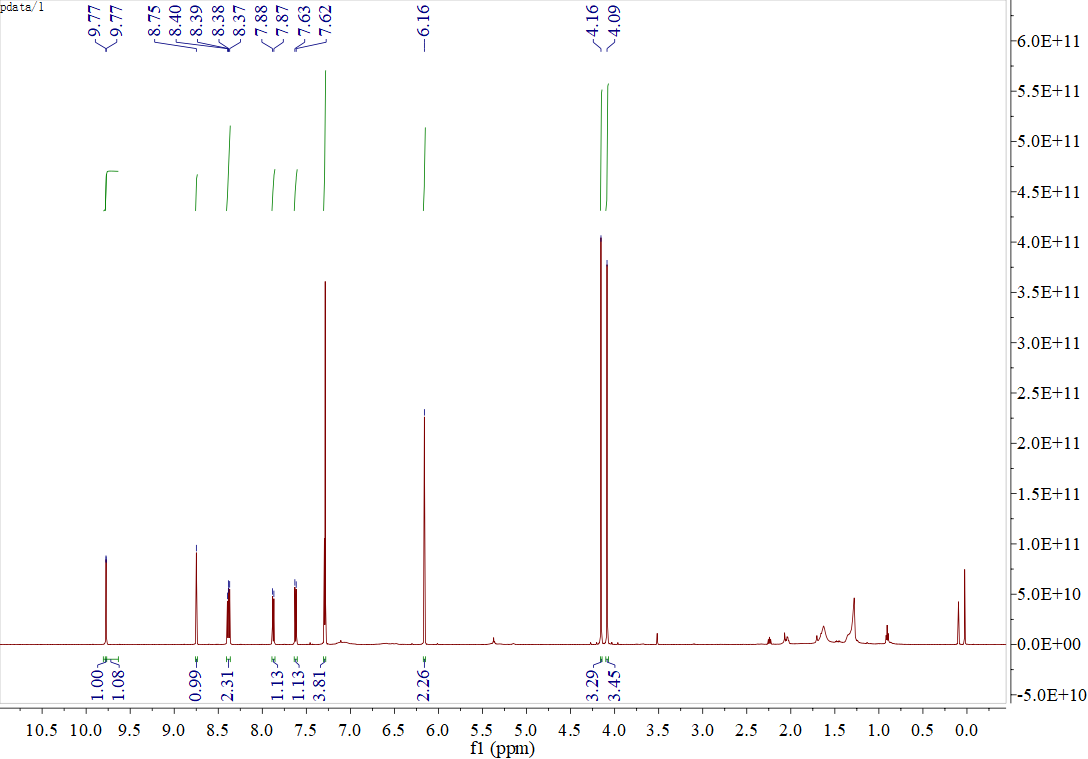


**Fig SS13.** ^1^H-NMR spectrum of compound **11** in CDCl_3_ (600 MHz).


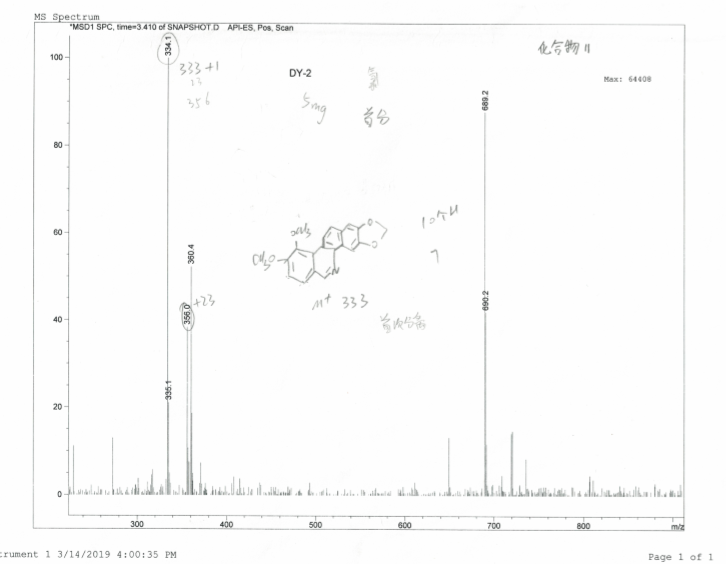


**Fig SS14.** ESI-MS spectrum of compound **11**.


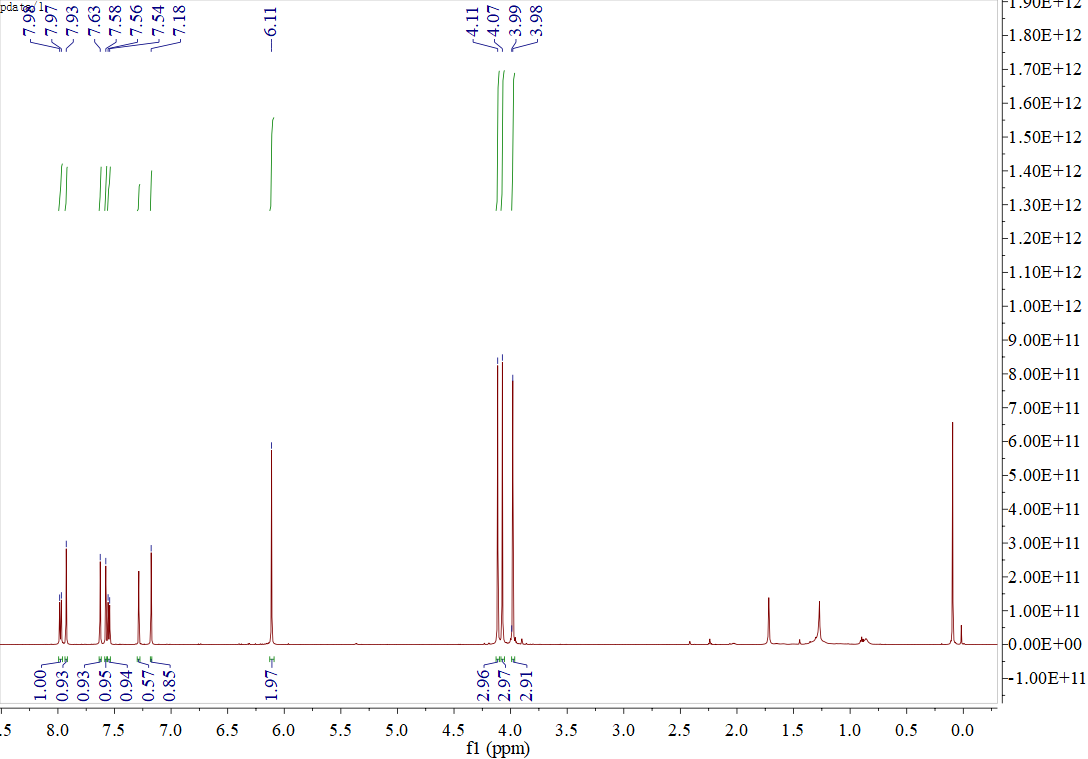


**Fig SS15.** ^1^H-NMR spectrum of compound **12** in CDCl_3_ (600 MHz).


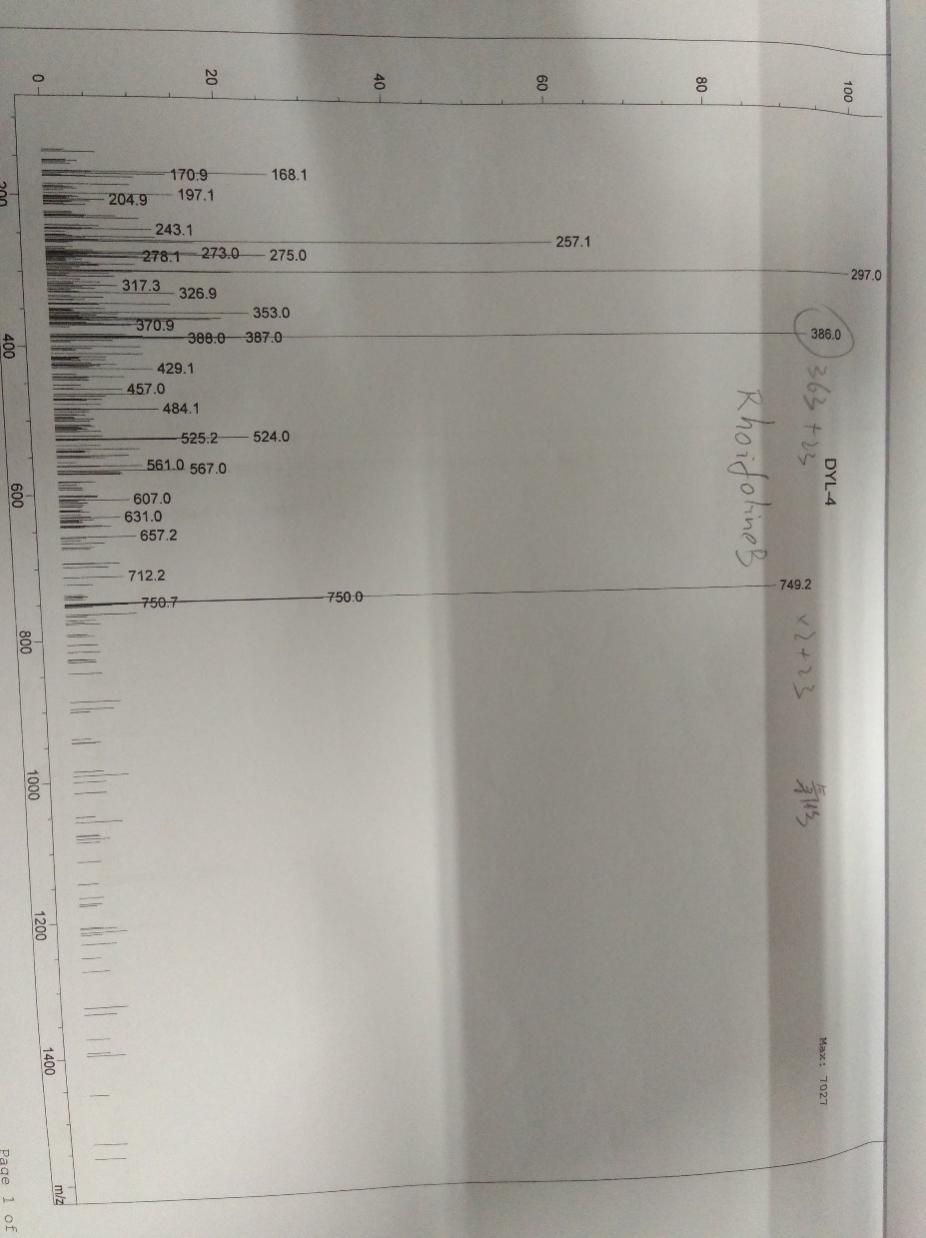


**Fig SS16.** ESI-MS spectrum of compound **12**.


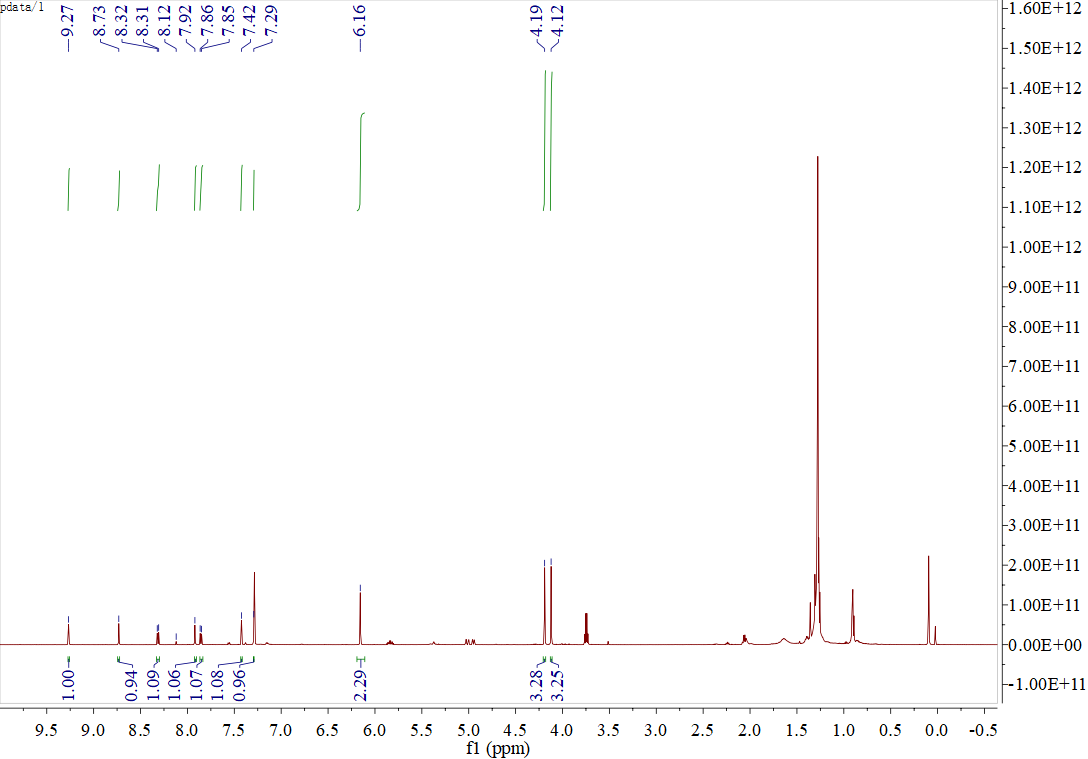


**Fig SS17.** ^1^H-NMR spectrum of compound **13** in CDCl_3_ (600 MHz).


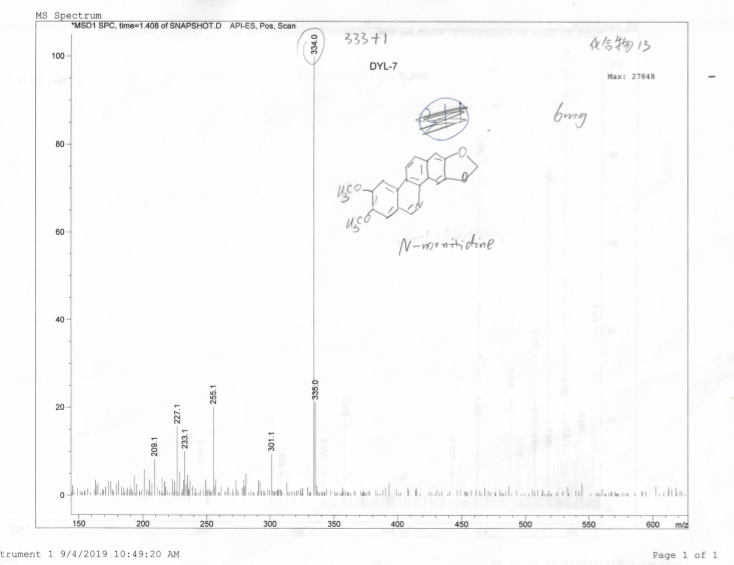


**Fig SS18.** ESI-MS spectrum of compound **13**.


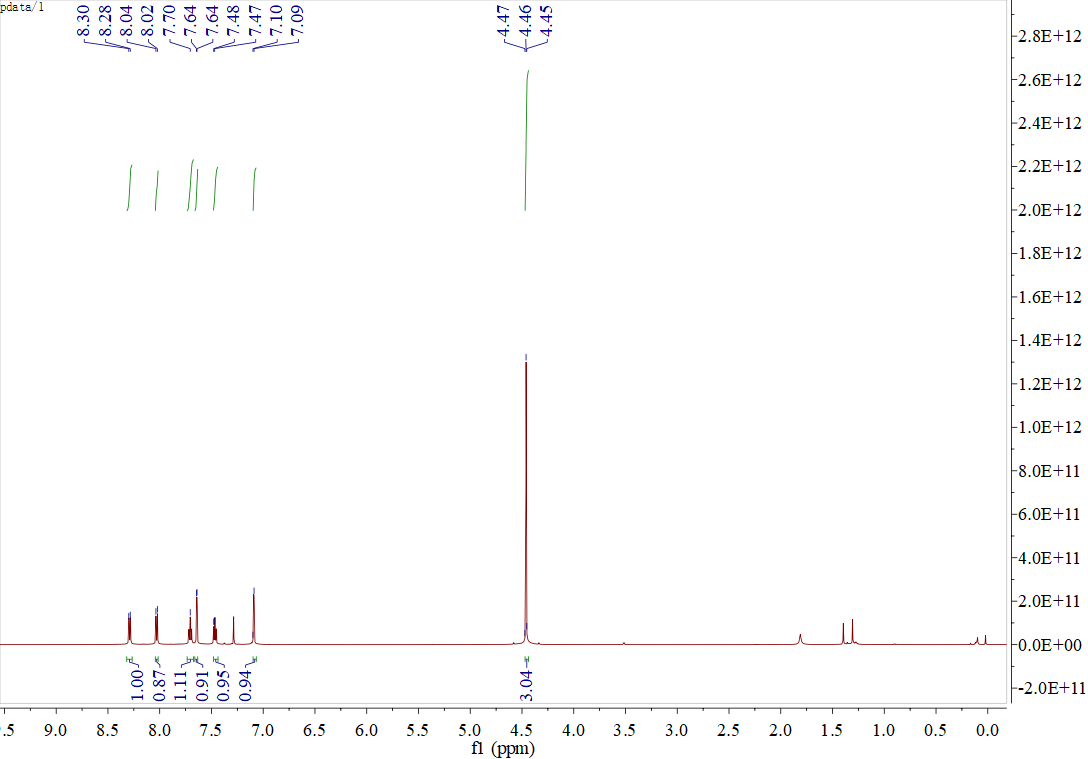


**Fig SS19.** ^1^H-NMR spectrum of compound **17** in CDCl_3_ (600 MHz).


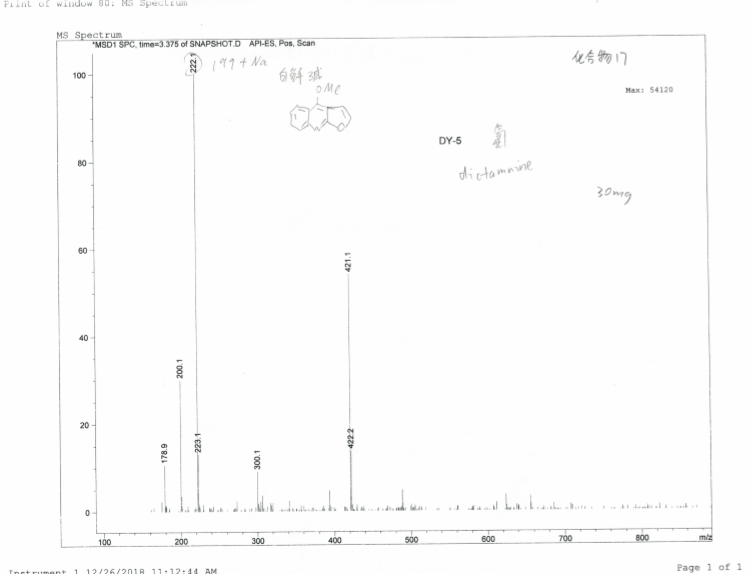


**Fig SS20.** ESI-MS spectrum of compound **17**.


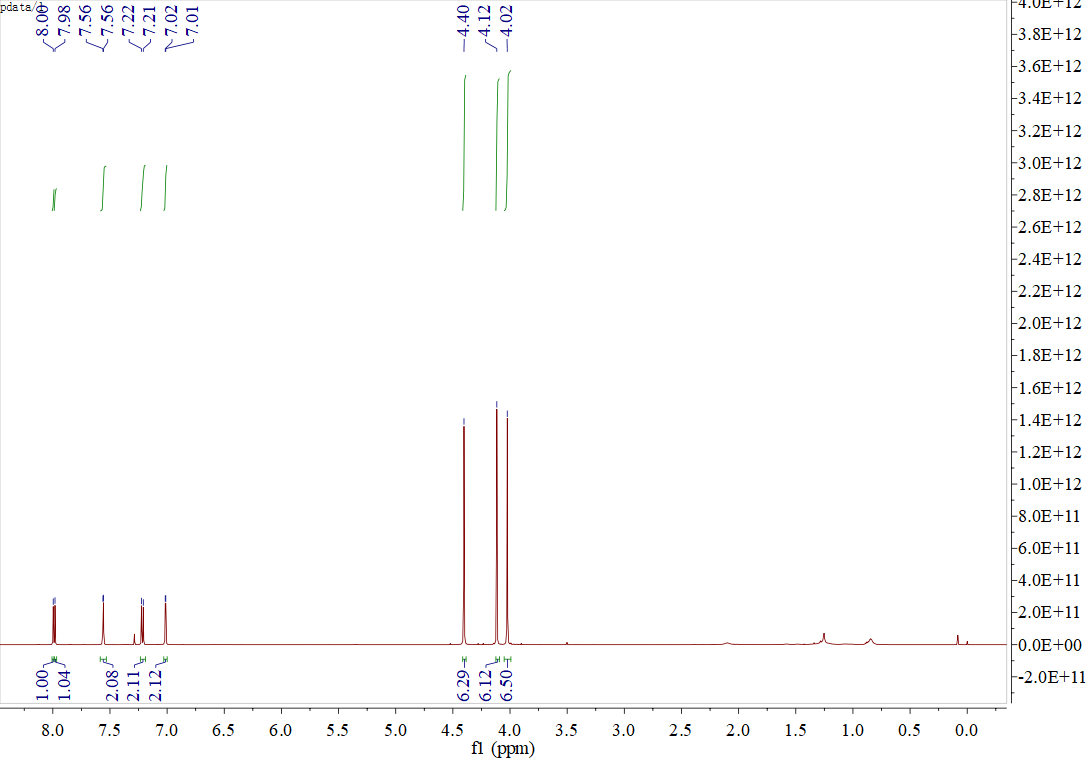


**Fig SS21.** ^1^H-NMR spectrum of compound **19** in CDCl_3_ (600 MHz).


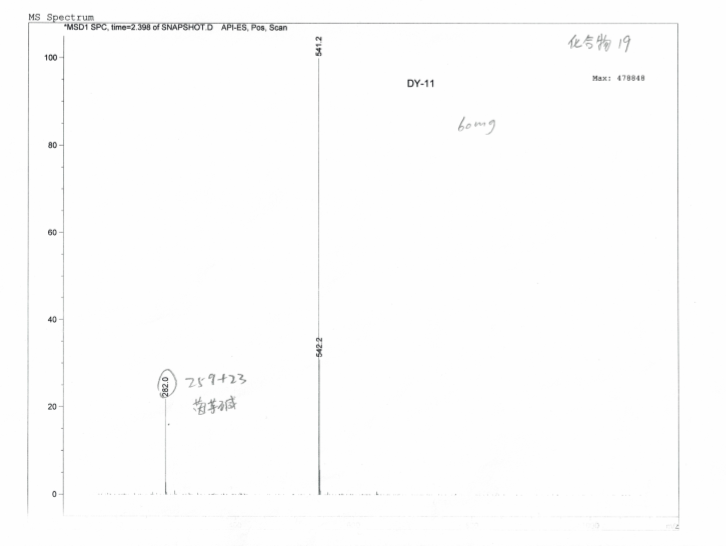


**Fig SS22.** ESI-MS spectrum of compound **19**.


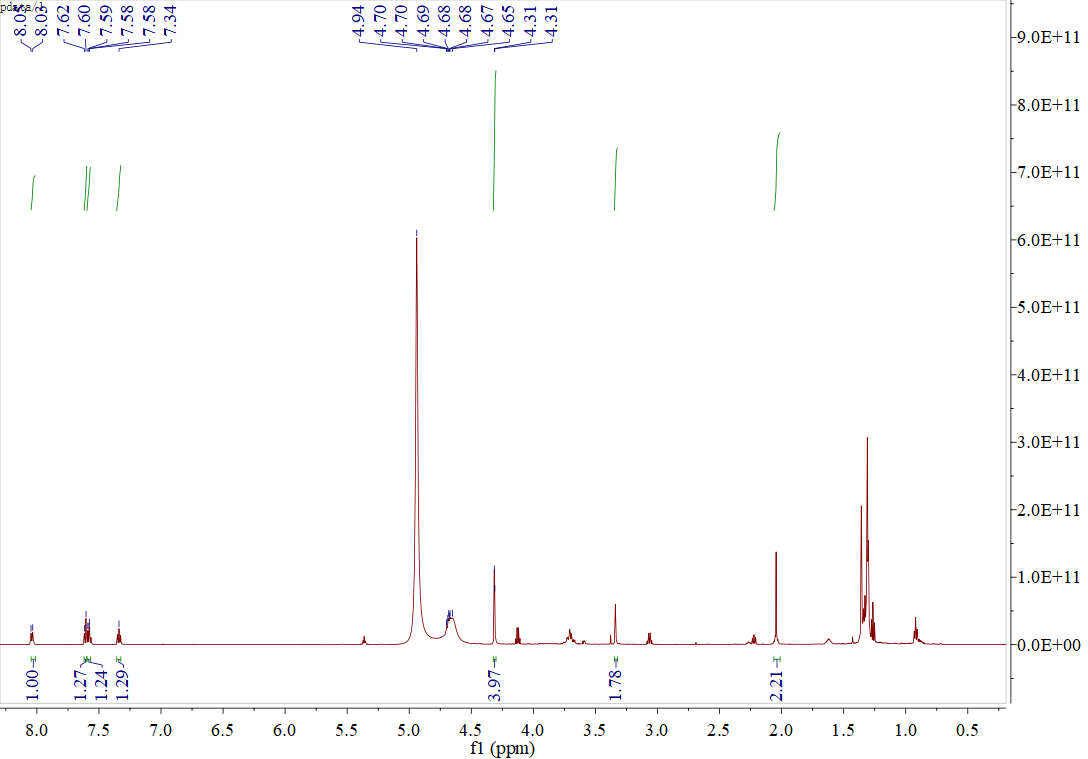


**Fig SS23.** ^1^H-NMR spectrum of compound **21** in CDCl_3_ (600 MHz).


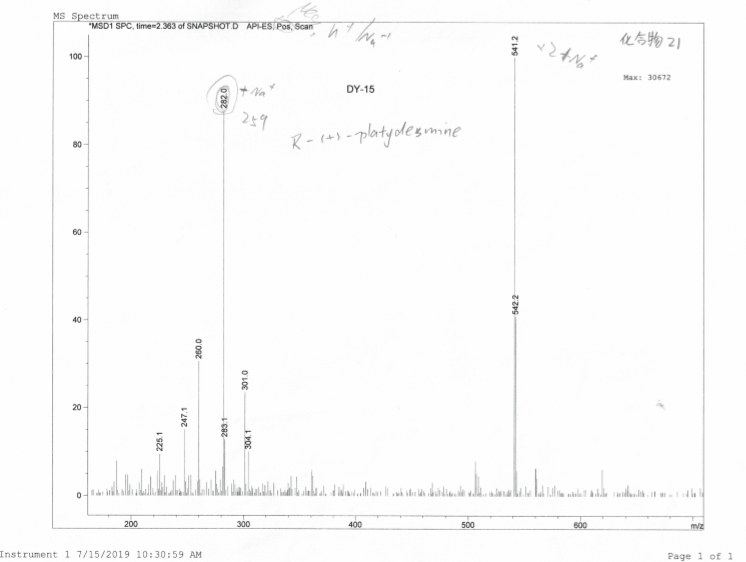


**Fig SS24.** ESI-MS spectrum of compound **21**.


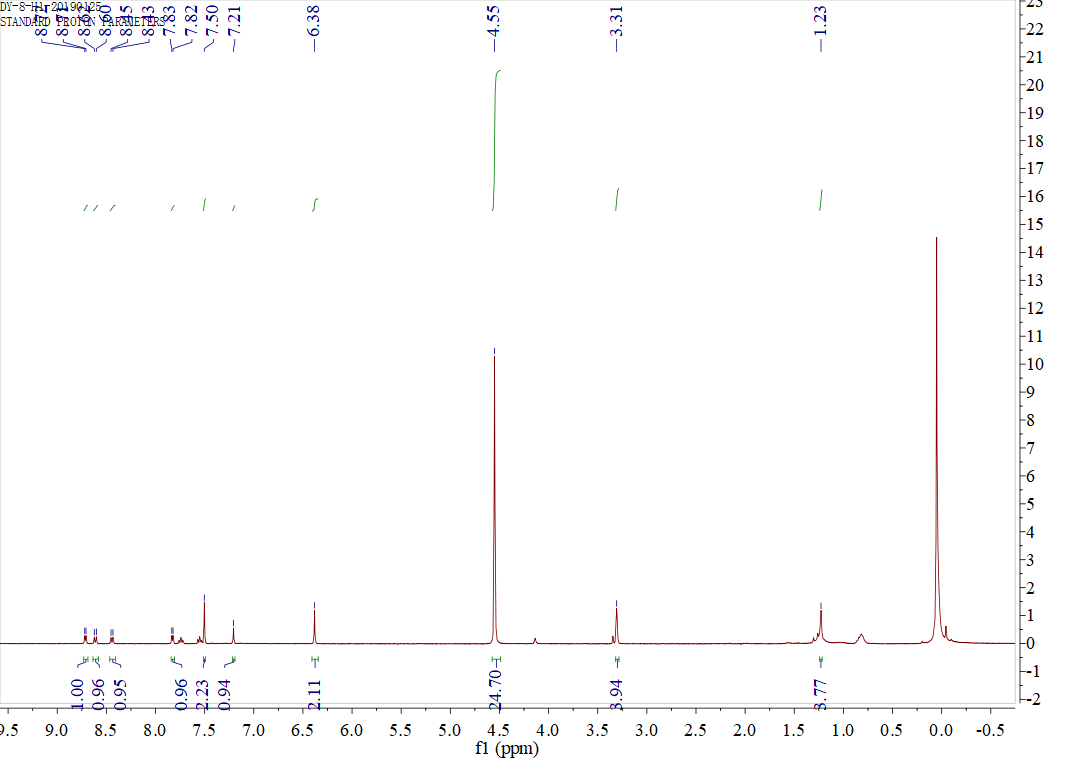


**Fig SS25.** ^1^H-NMR spectrum of compound **24** in CDCl_3_ (600 MHz).


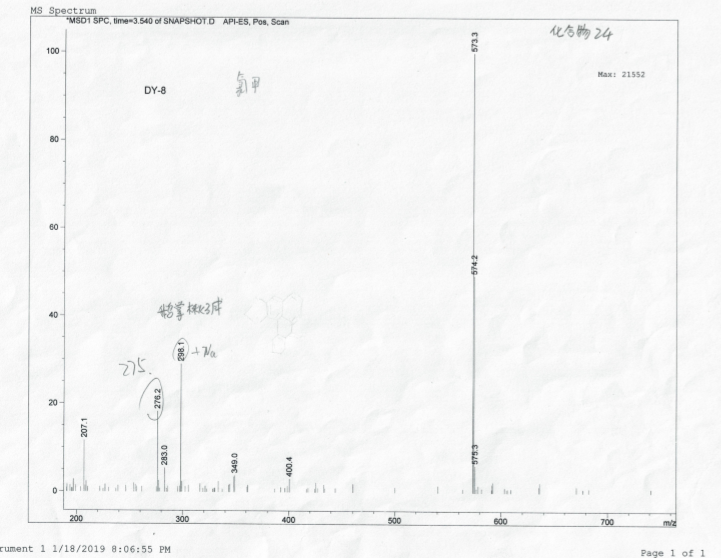


**Fig SS26.** ESI-MS spectrum of compound **24**.
